# A Unique Sulfotransferase-Involving Strigolactone Biosynthetic Route in Sorghum

**DOI:** 10.3389/fpls.2021.793459

**Published:** 2021-12-14

**Authors:** Sheng Wu, Yanran Li

**Affiliations:** Department of Chemical and Environmental Engineering, University of California, Riverside, Riverside, CA, United States

**Keywords:** strigolactones, sorghum, sulfotransferase, biosynthesis, synthetic biology

## Abstract

LOW GERMINATION STIMULANT 1 (LGS1) plays an important role in strigolactones (SLs) biosynthesis and *Striga* resistance in sorghum, but the catalytic function remains unclear. Using the recently developed SL-producing microbial consortia, we examined the activities of sorghum MORE AXILLARY GROWTH1 (MAX1) analogs and LGS1. Surprisingly, SbMAX1a (cytochrome P450 711A enzyme in sorghum) synthesized 18-hydroxy-carlactonoic acid (18-hydroxy-CLA) directly from carlactone (CL) through four-step oxidations. The further oxidated product orobanchol (OB) was also detected in the microbial consortium. Further addition of LGS1 led to the synthesis of both 5-deoxystrigol (5DS) and 4-deoxyorobanchol (4DO). Further biochemical characterization found that LGS1 functions after SbMAX1a by converting 18-hydroxy-CLA to 5DS and 4DO possibly through a sulfonation-mediated pathway. The unique functions of SbMAX1 and LGS1 imply a previously unknown synthetic route toward SLs.

## Introduction

**FIGURE 1:**
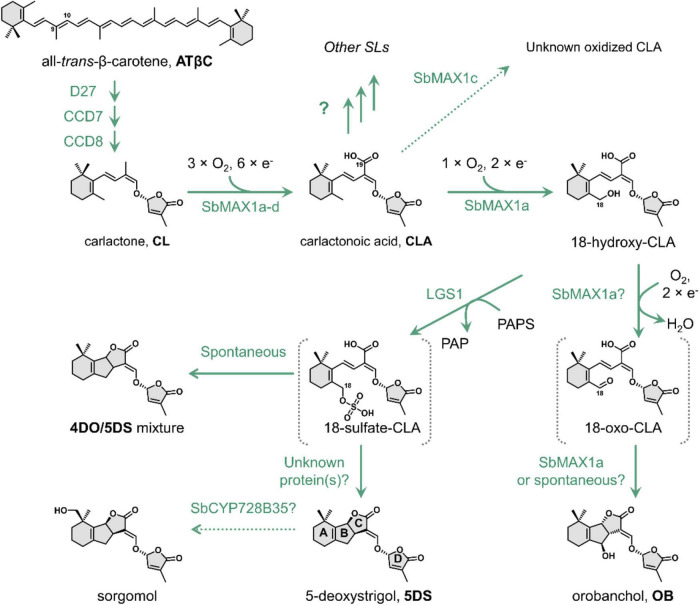
The proposed biosynthetic pathway of 5DS and OB in *Sorghum bicolor*. D27, [2Fe-2S]-containing isomerase DWARF27. Abbreviations: CCD7, carotenoid cleavage dioxygenase 7; CCD8, carotenoid cleavage dioxygenase 8; SbMAX1a, MAX1 analog a from *S. bicolor*; LGS1, *LOW GERMINATION STIMULANT 1*, a sulfotransferase; PAPS, 3′-phosphoadenosine 5′-phosphosulfate; PAP, 3′-phosphoadenosine-5′-phosphate; 4DO, 4-deoxyorobanchol; 5DS, 5-deoxystrigol.

Strigolactones (SL) are a group of butanolide-containing molecules originally identified as seed germination stimulants for the parasitic weeds *Striga* and *Orobanche* (Cook et al., 1966; Samejima et al., 2016) and later characterized as phytohormones that play diverse important roles in plant growth and development (Al-Babili and Bouwmeester, 2015; Zwanenburg and Blanco-Ania, 2018; Chesterfield et al., 2020). SLs can be divided into canonical and non-canonical SLs, with canonical SLs further grouped into strigol (*S*)- and orobanchol (OB) (*O*)-type SLs according to the stereochemistry of the C-ring (Al-Babili and Bouwmeester, 2015; Figure 1). Different SL structures have been reported to exhibit distinct parasitic weed germination activities (Yoneyama et al., 2010; Zwanenburg and Pospisil, 2013). For example, SLs exhibiting high germination stimulation activity toward *S. gesnerioides* induced low germination in *S. hermonthica*, while several SLs of high germination stimulation activity to *S. hermonthica* inhibit the germination of *S. gesnerioides* (Nomura et al., 2013). Recently, LOW GERMINATION STIMULANT 1 (LGS1) has been identified to be responsible for the *Striga* germination stimulant activity in sorghum and missing from the *Striga*-resistant sorghum varieties (Gobena et al., 2017), which produce distinct SL profiles, i.e., (*S*)-type 5-deoxystrigol (5DS) and (*O*)-type OB, respectively (Gobena et al., 2017). LGS1 is a putative sulfotransferase (SOT), which normally catalyzes the transfer of a sulfonate group from 3′-phosphoadenosine 5′-phosphosulfate (PAPS) to a hydroxyl group of acceptor molecules (Paul et al., 2012). The mechanism of how LGS1 regulates SL profiles between 5DS and OB in sorghum remains unclear.

**FIGURE 2:**
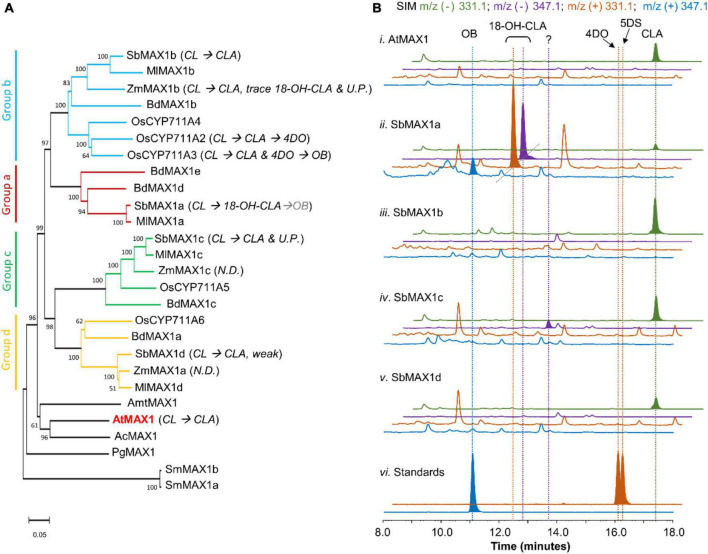
Functional characterization of MAX1 analogs from *S. bicolor*. **(A)** Phylogenetic analysis of MAX1 analogs. The phylogenetic tree was reconstructed in MEGA X using the neighbor-joining method based on amino acid sequence. The MAX1 analogs are from dicotyledons and monocotyledons. Species abbreviations: Sb, *Sorghum biocolor*; Ml, *Miscanthus lutarioriparius*; Zm, *Zea mays*; Bd, *Brachypodium distachyon*; Os, *Oryza sativa*; Amt, *Amborella trichopoda*; At, *Arabidopsis thaliana*; Ac, *Aquilegia coerulea*; Pg, *Picea glauca*; Sm, *Selaginella moellendorffii*. For the accession numbers of proteins, see Supplementary Table 6. UP, Unknown product; ND, no detected. **(B)** SIM extracted ion chromatogram (EIC) at *m/z*^–^ = 331.1 (green), 347.1 (purple), and *m/z*^+^ = 331.1 (orange), 347.1 (blue) of CL-producing *E. coli* co-cultured with *A. thaliana* P450 reductase 1 (ATR1)-expressing yeast (i) expressing AtMAX1, (ii–v) expressing SbMAX1a–d, and (vi) standards of OB, 4DO, and 5DS. CLA shows characteristic *m/z*^–^ = 331.1 (*MW* = 332.40, [C_19_H_24_O_5_-H]^–^ = [C_19_H_23_O_5_]^–^ = 331.1); 18-hydroxy-CLA shows characteristic *m/z*^–^ = 347.1 and *m/z*^+^ = 331.1 (*MW* = 348.40, [C_19_H_24_O_6_-H]^–^ = [C_19_H_23_O_6_]^–^ = 347.1, [C_19_H_24_O_6_-H_2_O + H]^+^ = [C_19_H_23_O_5_]^+^ = 331.1); OB shows characteristic *m/z*^+^ = 347.1 (*MW* = 346.38, [C_19_H_22_O_6_ + H]^+^ = [C_19_H_23_O_6_]^+^ = 347.1); 4DO and 5DS show characteristic *m/z*^+^ signal (*MW* = 330.38, [C_19_H_22_O_5_ + H]^+^ = [C_19_H_23_O_5_]^+^ = 331.1). All the traces are representative of at least three biological replicates for each engineered *E. coli*-*S. cerevisiae* consortium. 18-OH-CLA stands for 18-hydroxy-CLA. *MW* stands for molecular weight. Strain used for analysis: AtMAX1 (ECL/YSL1, Supplementary Table 3), SbMAX1a-d (ECL/YSL2a–d, Supplementary Table 3).

Strigolactones are synthesized from carlactone (CL), which is then converted to diverse SL structures by various downstream tailoring enzymes especially cytochrome P450s (CYPs) (Figure 1; Wang and Bouwmeester, 2018; Chesterfield et al., 2020). The two major groups of CYP that contribute to the structural diversity downstream of CL belong to CYP711A and CYP722C subfamily (Nelson et al., 2008). The best studied CYP711A is MORE AXILLARY GROWTH1 (MAX1) from *Arabidopsis thaliana* (AtMAX1), which converts CL to carlactonoic acid (CLA) and is functionally conserved in dicots (Challis et al., 2013). On the other hand, monocots, especially the economically significant *Poaceae* family, often encode more than one CYP711As (Supplementary Table 1; Figure 2A; Supplementary Figure 1), with diverse functions distinct from AtMAX1 (Challis et al., 2013; Zhang et al., 2014; Marzec et al., 2020; Changenet et al., 2021). For example, rice has five MAX1 homologs, with CYP711A2 catalyzing the conversion of CL to 4-deoxyorobanchol (4DO) and CYP711A3 further oxidizing 4DO to OB (Zhang et al., 2014). Most CYP711As encoded by monocot plants remain to be characterized. The other major group of SL-synthesizing CYPs, CYP722C subfamily, catalyzes the conversion of CLA toward either OB or 5DS (Wakabayashi et al., 2019, 2020; Wu et al., 2021). Currently, there are two known routes toward the synthesis of (*O*)-type SLs catalyzed by either group I CYP722C (e.g., VuCYP722C) or OsCYP711A2 (Zhang et al., 2014; Wakabayashi et al., 2019), while the only known 5DS biosynthetic route is through group II CYP722C (e.g., GaCYP722C) (Wakabayashi et al., 2020). However, CYP722Cs are generally missing from the *Poaceae* family including sorghum, which implies that sorghum employs a previously unknown strategy to synthesize (*S*)-type SL.

In this study, harnessing the recently developed SL-producing microbial consortia (Wu et al., 2021; Supplementary Figure 2), we investigated SL biosynthesis in *Sorghum bicolor*, which turns out to be distinct from that in rice (Zhang et al., 2014). We identified SbMAX1a as a unique CYP that catalyzes up to four oxidation steps converting CL to 18-hydroxy-CLA and a small amount of OB. Following this discovery, we found the substrate of LGS1 is likely 18-hydroxy-CLA. The addition of sulfo group to 18-hydroxy-CLA can inhibit further oxidation toward the synthesis of OB and the putative intermediate 18-sulfate-CLA synthesized from LGS1 can spontaneously form comparable amount of 4DO and 5DS with sulfate functioning as an easier leaving group than the original hydroxyl. This study discovered a second synthetic route toward the synthesis of (*S*)-type SL, which employs the unique SOT LGS1. However, the enzyme catalyzing the exclusive conversion of 18-sulfate-CLA to 5DS is still missing and requires further investigation into sorghum (Figure 1). Out independent identification of LGS1 using SL-producing microbial consortium is consistent with the very recently published characterization of LGS1 heterologously in tobacco and *in vitro* (Yoda et al., 2021).

## Materials and Methods

### Reagents and General Procedures

(±)5-deoxystrigol (purity >98%) and (±)-OB were purchased from Strigolab (Torino, Italy). (±)4-deoxyorobanchol [also named as (±)-2′-epi-5DS] were purchased from Chempep Incorporation (Wellington, FL, United States). PAPS lithium salt hydrate and the antibiotics were purchased from Sigma-Aldrich Corporation (St. Louis, MO, United States). The BP Clonase II Enzyme Mix, LR Clonase II Enzyme Mix, and Gateway pDONR221 vector were obtained from Invitrogen (Carlsbad, CA, United States). The *Saccharomyces cerevisiae* (*S. cerevisiae*) Advanced Gateway Destination Vector Kit was obtained from Addgene (Watertown, MA, United States). Expand high-fidelity PCR system (Roche Life Science, Pleasanton, CA, United States) was used for PCR reactions (Bio-Rad, Hercules, CA, United States). The *Escherichia coli* (*E. coli*) top 10 competent cells were purchased from Life Technologies (Pleasanton, CA, United States). The genes were synthesized by Integrated DNA Technologies (Coralville, IA, United States) and primers were synthesized by Life Technologies (Pleasanton, CA, United States). DNA sequencing was performed at Genewiz (San Diego, CA, United States). All the plasmids and strains used in this study are shown in Supplementary Tables 2, 3.

For CL production, XY medium [13.3 g/l monopotassium phosphate (KH_2_PO_4_), 4 g/l diammonium phosphate [(NH_4_)_2_HPO_4_], 1.7 g/l citric acid, 0.0025 g/l cobalt(II) chloride (CoCl_2_), 0.015 g/l manganese(II) chloride (MnCl_2_), 0.0015 g/l copper(II) chloride (CuCl_2_), 0.003 g/l boric acid (H_3_BO_3_), 0.0025 g/l sodium molybdate (Na_2_MoO_4_), 0.008 g/l zinc acetate [Zn(CH_3_COO)_2_], 0.06 g/l iron(III) citrate, 0.0045 g/l thiamine, 1.3 g/l magnesium sulfate (MgSO_4_), 5 g/l yeast extract, and 40 g/l xylose, pH 7.0] was prepped and used as previously described (Wu et al., 2021). For yeast ectopic expression, synthetic dropout (SD) medium (SDM) was used [0.425 g yeast nitrogen base (YNB) (BD Biosciences, San Jose, CA, United States), 1.25 g ammonium sulfate [(NH_4_)_2_SO_4_] dissolved in 200 ml distilled water (dH_2_O), autoclave at 121°C for 20 min. Add 25 ml 200 g/l glucose and 25 ml 20 g/l amino acid drop-out mix (Takara Bio USA, Inc. Mountain View, CA, United States) solution to prepare the medium]. Liquid chromatography–mass spectrometry (LC-MS) was carried out on a Shimadzu LC-MS 2020 (Kyoto, Japan) with LC-MS grade solvent. High-resolution mass spectrometry (HR-MS) analysis was carried on a Synapt G2-Si quadrupole time-of-flight mass spectrometer (Waters, Milford, MA, United States) coupled to an I-class ultra-performance liquid chromatography (UPLC) system (Waters, Milford, MA, United States).

### Plasmid Construction

All the genes were codon optimized for *S. cerevisiae* (Supplementary Table 4), synthesized, and cloned into the entry vector pDONR221 (Invitrogen, Carlsbad, CA, United States) through Gateway^®^ BP reaction. The genes were then introduced to the yeast expression vector through Gateway^®^ LR reaction using destination vectors from the Yeast Gateway Kit (Alberti et al., 2007). LGS1 mutants were constructed through PCR using primers shown in Supplementary Table 5. PCR was performed using pAG416GPD-LGS1 as the template with expand high-fidelity PCR system. The amplified DNA fragment was purified, recovered, and used to construct the expression plasmid with Gibson assembly.

### Culture Conditions for *E. coli*-Yeast Consortium-Based Strigolactone Production

The *E. coli* strain ECL for CL production (Supplementary Table 3) was prepared as described previously (Wu et al., 2021). Single colony was grown overnight at 37°C in 1 ml Luria-Bertani (LB) containing 25 μg/ml chloramphenicol, 50 μg/ml spectinomycin, and 100 μg/ml ampicillin. 500 μL of the overnight culture was then used to inoculate 5 ml of fresh LB with the corresponding antibiotics and cultured at 37°C and 220 rpm in the 100 ml Erlenmeyer flask. When optical density 600 (*OD*_600_) reached ∼0.6, isopropyl β-D-1-thiogalactopyranoside (IPTG) was added with the final concentration at 0.2 mM, with ferrous sulfate supplemented at the same time (final concentration at 10 mg/l). Then, the cultures were incubated at 22°C and 220 rpm for 15 h. At the same time, single colony of each yeast strain harboring the corresponding cytochrome P450-expression constructs was used to inoculate 1 ml SDM. The seed culture was incubated at 28°C and 220 rpm overnight. 100 μl of the overnight grown seed culture was used to inoculate 5 ml of the corresponding SD medium in a 100-ml Erlenmeyer flask and grown at 28°C for 15 h. The *E. coli* and yeast cells were harvested by centrifugation at 3,500 rpm for 5 min. Then, they were resuspended together in 5 ml of XY media and grown at 22°C for 60 h.

### Metabolite Isolation and Analysis

Collect the cell pellet through centrifugation in a 50-ml centrifuge tube at 4,000 rpm for 10 min. The supernatant is transferred to another 50 ml centrifuge tube. The cell pellets were resuspended in 150 μl dimethylformamide (DMF) and 850 μl acetone, vortexed for 15 min, and centrifuged at 12,000 rpm for 10 min. The medium was extracted with 4 ml of ethyl acetate (EtOAc), vortex for 1 min, and centrifuged at 4,000 rpm for 20 min. The supernatant from both the portions was collected and transferred to 1.7 ml microcentrifuge tubes, dried in Vacufuge under reduced pressure (Eppendorf concentrator plus, Enfield, CT, United States), and redissolve in 100 μl acetone. The extract was centrifuged at 12,000 rpm for 10 min before *LC-MS* analysis. The *LC-MS* analysis were performed on a C18 column (Kinetex^®^ C18, 100 mm × 2.1 mm, 100 Å, particle size 2.6 μm; Phenomex, Torrance, CA, United States). For separation method I, the parameters were set as follows: column temperature: 40°C, flow rate: 0.4 ml/min; mobile phase A: water containing 0.1% (v/v) formic acid; mobile phase B: acetonitrile containing 0.1% (v/v) formic acid. The LC program was set as follows: 0–28 min, 5–100% B; 28–35 min, 100% B; 35–40 min, 5% B.

### Heterologous Expression of Recombinant LOW GERMINATION STIMULANT 1 in *S. cerevisiae*

We first expressed LGS1 from *E. coli* strain BL21 (DE3), but failed. Then, LGS1 was expressed from *S. cerevisiae* CEN.PK2-1D using low-copy number plasmid (pAG416GPD-LGS1, Supplementary Table 2). One fresh colony of the LGS1-expressing yeast strain was first cultured in 1 ml SDM lacking uracil (SD-Ura) medium, grown at 30°C, and 220 rpm for overnight in a shaker incubator. 100 μl of the overnight culture was used to inoculate 5 ml SD-Ura medium (*OD*_600_ ≈ 0.1), grown at 30°C, and 220 rpm for 48 h (*OD*_600_ ≈ 20). Cell pellets were then harvested by centrifuging at 3,500 rpm for 2 min, washed with 1 ml of water, and resuspended in 120 μl of 20 mM sodium phosphate buffer (*pH* = 7.4). 50 μl of silicon beads [0.5 mm, Research Products International (RPI, Mount Prospect, IL, United States)] were then added to the cell suspension, which is then chilled on ice, and lysed using cell disruptor (FastPrep^®^-24, MP Biomedicals, Irvine, CA, United States). The parameters were set as speed: 4.0 m/s and time: 30 s. The homogenate was centrifuge at 13,000 rpm for 2 min and the supernatant was used for the crude lysate-based enzyme assays.

### Yeast Crude Lysate-Based Enzyme Assays

50 μl of crude enzyme extract mentioned above is incubated with 5 μl of concentrated metabolic extract dissolved in DMF (extracted from 3 ml co-culture strain), with or without 100 μM PAPS, and incubated at 30°C for 1 h. Enzyme assay using yeast strain expressing an empty vector as the negative control. The reaction mixture was quenched by adding an equal volume of acetonitrile followed by vigorous vortexing to remove the protein. The quenched reaction mixtures were then centrifuged at 13,000 rpm for 10 min. 17 μl of supernatant was subjected to *LC-MS* analysis with the C18 column (Kinetex^®^ C18, 100 mm × 2.1 mm, 100 Å, particle size 2.6 μm; Phenomex, Torrance, CA, United States). To detect putative 18-sulfate-CLA, an intermediate with an increased polarity, we use a different separation method: Separation Method II. The parameters were set as follows: column temperature: 25°C, flow rate: 0.4 ml/min; mobile phase A: water containing 0.1% (v/v) formic acid; mobile phase B: acetonitrile containing 0.1% (v/v) formic acid. The LC program was set as follows: 0–3 min, 5–11% B; 3–13 min, 11–19% B; 13–21 min, 19–27.5% B; 21–24 min, 27.5–34% B; 24–28 min, 34–42% B; 28–32 min, 42–90% B; 32–34 min, 90–100% B; 34–35.5 min, 100–5% B; 35.5–40 min, 5% B.

## Results and Discussion

### Functional Mapping of Sorghum MORE AXILLARY GROWTH1 Analogs in Carlactone-Producing Microbial Consortium

Same as the other *Poaceae* family members, sorghum does not encode CYPs that belong to CYP722C subfamily, but encode four MAX1 analogs. To understand the evolutionary relationship of these MAX1 homologs, we conducted a phylogenetic analysis of selected MAX1 analogs from dicotyledons and monocotyledons (Figure 2A; Supplementary Figure 1; Supplementary Table 6). Noticeable, the MAX1 analogs from grasses fall into four different subclades, which are named group a-d here for simplicity (Figure 2A). Four MAX1 analogs of sorghum fall into each of the four groups, while maize and rice only encode MAX1 analogs from group b-d but not group a. To understand the biosynthetic machinery of 5DS and OB in sorghum, MAX1 analogs from sorghum (Supplementary Table 1) were introduced to the CL-producing microbial consortia (ECL, Supplementary Table 3; Figure 2B). Interestingly, expression of SbMAX1a to CL-producing consortium (ECL/YSL2a, Supplementary Table 3) led to the synthesis of OB and 18-hydroxy-CLA [verified through high-resolution mass spectrometry (HR-MS) analysis, Supplementary Figure 3A]. The production of 18-hydroxy-CLA by SbMAX1a is much more efficient than all the SL synthetic CYPs we examined previously (CYP722Cs and OsCYP711A2, resulting in ECL/YSL3-5, Supplementary Table 3; Figure 2B; Supplementary Figure 4; Wakabayashi et al., 2019). Likely SbMAX1a first catalyzes three-step oxidation on C19 to synthesize CLA, followed by additional oxidations on C18 to afford the synthesis of 18-hydroxy-CLA and subsequently 18-oxo-CLA, which than converts to OB (Figure 1; Wakabayashi et al., 2019; Mori et al., 2020). This result is partially consistent with the very recent characterization of SbMAX1a as an 18-hydroxy-CLA synthase, except for the detection of OB as a side product in ECL/YSL2a (Yoda et al., 2021). The conversion from 18-hydroxy-CLA to OB is catalyzed by SbMAX1a as shunt product or by endogenous enzymes in yeast or *E. coli* that remains to be investigated.

In addition, SbMAX1c converted CL to CLA and one new peak of molecular weight same as 18-hydroxy-CLA (16 Da more than that of CLA) (Figure 2B and Supplementary Figure 3B). However, due to the low titer of SLs from the microbial consortia and the lack of commercially available standards, we cannot verify the identities of this compound synthesized by SbMAX1c currently. The failure to clearly characterize the function of SbMAX1c demonstrates the importance to enhance SL production of this microbial consortium as a useful tool in SL biosynthesis characterization. The other two MAX1 analogs examined simply catalyze the conversion of CL to CLA without further structural modifications (Figure 2B). The MAX1 analogs were also introduced to ECL/YSL2a or ECL/YSL5 that produce 18-hydroxy-CLA and OB or 5DS (resulting strain: ECL/YSL6-7, Supplementary Table 3), but no new conversions were detected (Supplementary Figure 5). The newly discovered and unique activities of SbMAX1a and SbMAX1c imply the functional diversity of MAX1 analogs encoded by monocot plants, with much remains to be investigated.

### LOW GERMINATION STIMULANT 1 Converts 18-Hydroxy-Carlactonoic Acid to 5-Deoxystrigol and 4-Deoxyorobanchol

**FIGURE 3:**
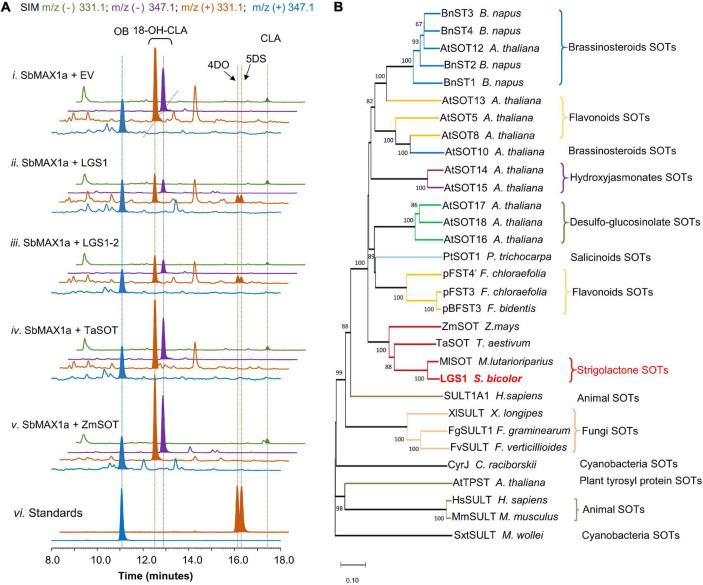
Functional characterization of LGS1 and analogs using CL-producing microbial consortium expressing SbMAX1a. **(A)** SIM EIC at *m/z*^–^ = 331.1 (green), 347.1 (purple), and *m/z*^+^ = 331.1 (orange), 347.1 (blue) of CL-producing *E. coli* co-cultured with yeast expressing ATR1, SbMAX1a and (i) empty vector (EV), (ii) LGS1, (iii) LGS1-2, (iv) sulfotransferase (SOT) from *Triticum aestivum* (TaSOT), (v) SOT from *Zea mays* (ZmSOT), and (vi) standards of OB, 4DO, and 5DS. All traces are representative of at least three biological replicates for each engineered *E. coli*-*S. cerevisiae* consortium. **(B)** Phylogenetic analysis of LGS1. The phylogenetic tree was reconstructed in MEGA X using the neighbor-joining method based on amino acid sequence. The SOTs are from animals, plants, fungi, and cyanobacteria. For the accession numbers of proteins, see Supplementary Table 7. The sequence of LGS1 is from sorghum WT Shanqui Red, LGS1-2 variation is a reference sequence from NCBI, and is four amino acids (DADD) longer than LGS1, see Supplementary Table 4.

While wild-type sorghum encoding *lgs1* (such as Shanqui Red) generally produce 5DS and a small amount of OB, the *lgs1* loss-of-function variants (such as SRN39) only produce OB but not 5DS (Gobena et al., 2017). Therefore, it has been suggested that LGS1 may play an essential role in regulating SL synthesis toward 5DS or OB in sorghum (Gobena et al., 2017). 18-hydroxy-CLA has been identified as a general precursor to the synthesis of canonical SL such as 4DO, 5DS, and OB (Zhang et al., 2014; Wakabayashi et al., 2019, 2020). Since the amount of 18-hydroxy-CLA is substantially higher in the *lgs1* mutant compared with the wild-type sorghum (Yoda et al., 2021), it is likely that LGS1 also employs 18-hydroxy-CLA as the substrate. LGS1 contains sulfotransferase (SOT) domain and may sulfate 18-hydroxy-CLA, similar to as some plant SOTs sulfate phytohormones [e.g., AtSOT10 sulfate brassinosteroids and AtSOT15 sulfate jasmonates (Hirschmann et al., 2014; Figure 3B)]. To synthesize 5DS by group II CYP722C (or 4DO by OsCYP711A2), likely C19 functions as the nucleophile to attack C18, which enables C18-hydroxy to recruit one proton and form water as the leaving group (Supplementary Figure 6; Zhang et al., 2014; Wakabayashi et al., 2020). However, the hydroxy group is generally not a favorable leaving group and it often needs to be activated to trigger the subsequent reactions (e.g., intramolecular cyclization). Common hydroxy activation strategies used in nature include acetylation, phosphorylation, and sulfonation (Muller et al., 2010; Chen et al., 2018; Yue et al., 2020). Sulfation/intramolecular cyclization has been reported to be employed in microbial natural product biosynthesis such as ficellomycin from *Streptomyces ficellus* (Yue et al., 2020), but seldom in plant. The discovery of the unique SbMAX1a synthesizing 18-hydroxy-CLA as the major product leads to the hypothesis that LGS1 may modify the 18-hydroxyl group to form 18-sulfate-CLA, which will prohibit further oxidation toward the formation of OB and promote the nucleophilic attack on C18 to form C ring.

Introduction of LGS1 to ECL/YSL2a (resulting ECL/YSL8a, Supplementary Table 3) resulted in substantial decrease of 18-hydroxy-CLA and the appearance of 4DO and 5DS (ratio ∼ 1:1, Figure 3A), though the amount is low in comparison to 18-hydroxy-CLA and OB (Figure 3A). This result is also consistent with the very recently reported characterization of LGS1 in converting 18-hydroxy-CLA to 5DS and 4DO in both the tobacco transient expression and *in vitro* assays (Yoda et al., 2021). Similar to many previous SOT studies (Hirschmann et al., 2014), the putative intermediate 18-sulfate-CLA was not detected from *in vivo* assays using SL-producing microbial consortia (Supplementary Figure 7). 4DO and 5DS are synthesized in similar levels, which indicate that the conversion from 18-sulfate-CLA to the canonical SL structures is likely spontaneous with 18-sulfate as an easier leaving group than water formed from 18-hydroxy (Supplementary Figure 8). There is likely other enzyme(s) involved downstream of or simultaneous with LGS1 to guarantee the conversion of 18-sulfate-CLA to 5DS exclusively instead of a 4DO/5DS mixture in sorghum. We, thus, examined the function of SbMAX1b-1d, SbCYP722B, SbCYP728B35, SbCYP728B1, and ZmCYP728B35 in the 4DO/5DS/18-hydroxy-CLA-producing consortium ECL/YSL8a (resulting ECL/YSL9-10, Supplementary Table 3; Wakabayashi et al., 2021). However, we were unable to see any changes to the ratio between 5DS and 4DO (Supplementary Figure 9). Further, genomics-based analysis on sorghum is required to identify the missing components that are responsible for the inversion of the stereochemistry on the C ring.

### Biochemical Characterization of LOW GERMINATION STIMULANT 1 as an 18-Hydroxy-Carlactonoic Acid Sulfotransferase

**FIGURE 4:**
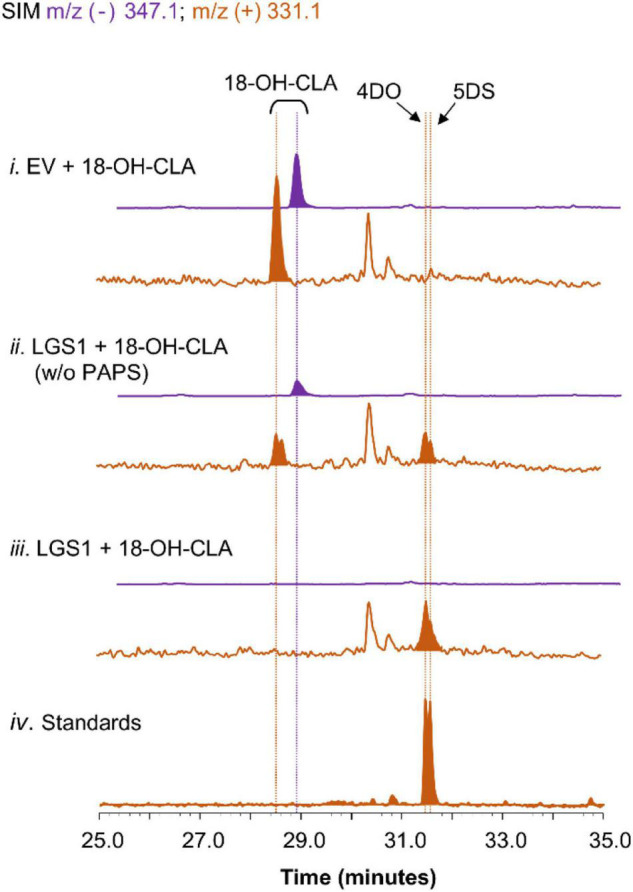
Characterization of LGS1 activity using crude lysate assay. SIM EIC at *m/z*^–^ = 347.1 (purple) and *m/z*^+^ = 331.1 (orange) of crude lysate assay using (i) EV-harboring yeast with PAPS, (ii) LGS1-expressing yeast without PAPS, (iii) LGS1-expressing yeast and PAPS, (iv) authentic standard of 4DO and 5DS. The reaction was incubated for 1 h with extracts of ECL/YSL2a medium and the samples were analyzed using separation method II (extraction method see section “Materials and Methods”).

To further validate the proposed mechanism of LGS1 in sorghum SL biosynthesis (Supplementary Figure 8), lysates from yeast expressing LGS1 were incubated with spent medium of CL-producing consortia expressing SbMAX1a. When LGS1 was assayed with 18-hydroxy-CLA and PAPS, 18-hydroxy-CLA was nearly completely consumed. 4DO and 5DS were observed, but not 18-sulfate-CLA, which is likely due to the low stability (Figure 4). The addition of PAPS to the lysate assay system results in enhanced consumption of 18-hydrxoy-CLA and also synthesis in 4DO/5DS (Figure 4), which indicates that LGS1 is a PAPS-dependent SOT.

Like other plant SOTs, LGS1 is predicted to be localized in cytoplasm. Cytosolic SOTs contain several conserved PAPS-binding motifs, including the one interacts with 5′-phosphate of PAPS (TYPKSGT), 3′-phosphate of PAPS (YxxRNxxDxxVS), and nucleotide of PAPS (GxxGxxK/R) (Xie et al., 2020). Multiple sequence alignment indicates that LGS1 contains these motifs, but with some variations (SLPKSGT and YxxRExxDxxVS, respectively) (Supplementary Figure 10). LGS1 contains the highly conserved histidine residues (H216) (Landi and Esposito, 2020) and moderately conserved histidine residues (H317A) (Supplementary Figure 10), which likely act as a base to remove the proton from the substrate hydroxyl group, thereby forming an oxygen anion, and then attacking the sulfo group of PAPS to complete the transfer of the sulfo group. To determine whether these residues play a key role in catalysis, we conducted site-directed mutagenesis on residues likely act as a catalytic base (H216A, H317A) or crucial for PAPS binding (K148A, Y247F) (Xie et al., 2020). While LGS1^*H*216*A*^ (resulting strain: YSL8f, Supplementary Table 3) exhibited same activity as wild type LGS1, replacing LGS1 with LGS1^*K*148*A*^, LGS1^*Y*247*F*^, and LGS1^*H*317*A*^ in ECL/YSL8a (resulting strain: YSL8g-i, Supplementary Table 3) completely abolished the synthesis of 4DO and 5DS (Supplementary Figure 11), implying that these residues are critical to the catalytic activity of LGS1 (Supplementary Figure 11).

### LOW GERMINATION STIMULANT 1-Mediated Strigolactone Biosynthesis Is Unique Among Characterized Sulfotransferases

Sulfotransferases universally exist in all the types of organisms and involve in the modification of both small molecules [e.g., steroids (Marsolais et al., 2007)] and macromolecules [e.g., glycosaminoglycans (Kusche-Gullberg and Kjellén, 2003)]. Among various plant SOTs, the ones from *A. thaliana* are the most studied, with 10 out of 21 AtSOTs of known functions or substrates (Hirschmann et al., 2014; Chan et al., 2019). To examine if similar LGS1-involved SL biosynthetic mechanism exists in other plants, likely *Poaceae* plants, we used LGS1 protein sequence as a query to seek for LGS1 analogs (Supplementary Table 7). We were only able to find one SOT from *Miscanthus lutarioriparius* (*M. lutarioriparius*) (MlSOT, 401 a.a., 80% identity) of high similarity to LGS1 (452 a.a.), while the next few on the list is all quite different from LGS1. We selected a few SOTs that exhibit highest similarity to LGS1 including MlSOT, SOTs from *Triticum aestivum* (TaSOT, 345 a.a., 55% identity), and *Zea mays* (ZmSOT, 451 a.a., 53% identity) and tested the activity in ECL/YSL8c-e (Supplementary Table 3). As expected, only MlSOT was able to synthesize 5DS and 4DO, but with a much lower efficiency than LGS1 (Supplementary Figure 11), while ZmSOT and TaSOT did not change the SL production profile (Figure 3A). To further understand the evolutionary relationship between LGS1 and other plant SOTs, we constructed a phylogenetic analysis of various SOTs from plants, animals, bacteria, and fungi (Supplementary Table 7 and Figure 3B). As expected, LGS1 belongs to plant SOT family, but is distinct from other characterized plant SOTs (Hirschmann et al., 2014). LGS1 and MlSOT are located on a unique subbranch that is different from all the other plant SOTs (Figure 3B).

Multiple independent natural LGS1 loss-of-function varieties have been found in *Striga*-prevalent areas in Africa and are rare outside of *Striga*-prone region, which indicates that the lack of *lgs1* gene can adapt to weed parasitism (Bellis et al., 2020). *M. lutarioriparius* encodes four MAX1 analogs and each exhibits high similarity and corresponds to one of the four SbMAX1s (Miao et al., 2021). Because MlSOT also exhibits the same activity as LGS1, highly likely *M. lutarioriparius* harnesses the same LGS1-involving strategy and produces similar SL profiles to sorghum.

The lack of LGS1 paralogs in other crops (e.g., maize) implies that much remains to be characterized about SL biosynthesis in these economically significant plants. For example, maize has been reported to produce 5DS and non-classical SLs but not (*O*)-type SLs (Awad et al., 2006; Charnikhova et al., 2017, 2018). However, same as other members from the *Poaceae* family, maize does not encode CYP722C analogs. The lack of LGS1 functional paralog, thus, indicates that a different synthetic route toward 5DS remains to be uncovered from maize. The activities of MAX1 analogs from maize (Supplementary Table 1) were examined in different microbial consortia as well (ECL/YSL11, Supplementary Table 3). ZmMAX1b (Yoneyama et al., 2018) exhibited similar activity to SbMAX1c: in addition to converting CL to CLA, it produced trace amounts of 18-hydroxy-CLA and an unknown oxidated product as SbMAX1c (Supplementary Figure 12). ZmMAX1a and c showed no activity toward CL (Supplementary Figure 12). Our results suggest that the 5DS biosynthesis in maize likely requires unknown types of enzymes yet to be identified.

## Conclusion

In summary, the identification of SbMAX1s implies the functional diversity of MAX1 analogs encoded by monocots and the characterization of LGS1 uncovers a unique biosynthetic route toward canonical SLs in sorghum. In addition, this study shows that SL-producing microbial consortium is a useful tool in the investigation of SL biosynthesis and highlights the necessity to enhance the performance of the microbial production platform for the functional elucidation of unknown enzymes (e.g., SbMAX1c).

## Data Availability Statement

The datasets presented in this study can be found in online repositories. The names of the repository/repositories and accession number(s) can be found in the article/Supplementary Material.

## Author Contributions

Both authors conceived the project, designed the experiments, and wrote the manuscript. SW performed the experiments and analyzed the results.

## Conflict of Interest

YL and SW filed a provisional patent application on January 28, 2021, “Strigolactone-producing Microbes and Methods of Making and Using the Same,” US Provisional Application No. 63/142,801.

## Publisher’s Note

All claims expressed in this article are solely those of the authors and do not necessarily represent those of their affiliated organizations, or those of the publisher, the editors and the reviewers. Any product that may be evaluated in this article, or claim that may be made by its manufacturer, is not guaranteed or endorsed by the publisher.
