# Supplementary material for: A Unique Sulfotransferase-Involving Strigolactone Biosynthetic Route in Sorghum
Source: Front Plant Sci. 2021 Dec 14;12:793459. doi: 10.3389/fpls.2021.793459 (PMC8713700; doi:10.3389/fpls.2021.793459)
Supplement: Supplementary file 1 [file Data_Sheet_1.docx]

**
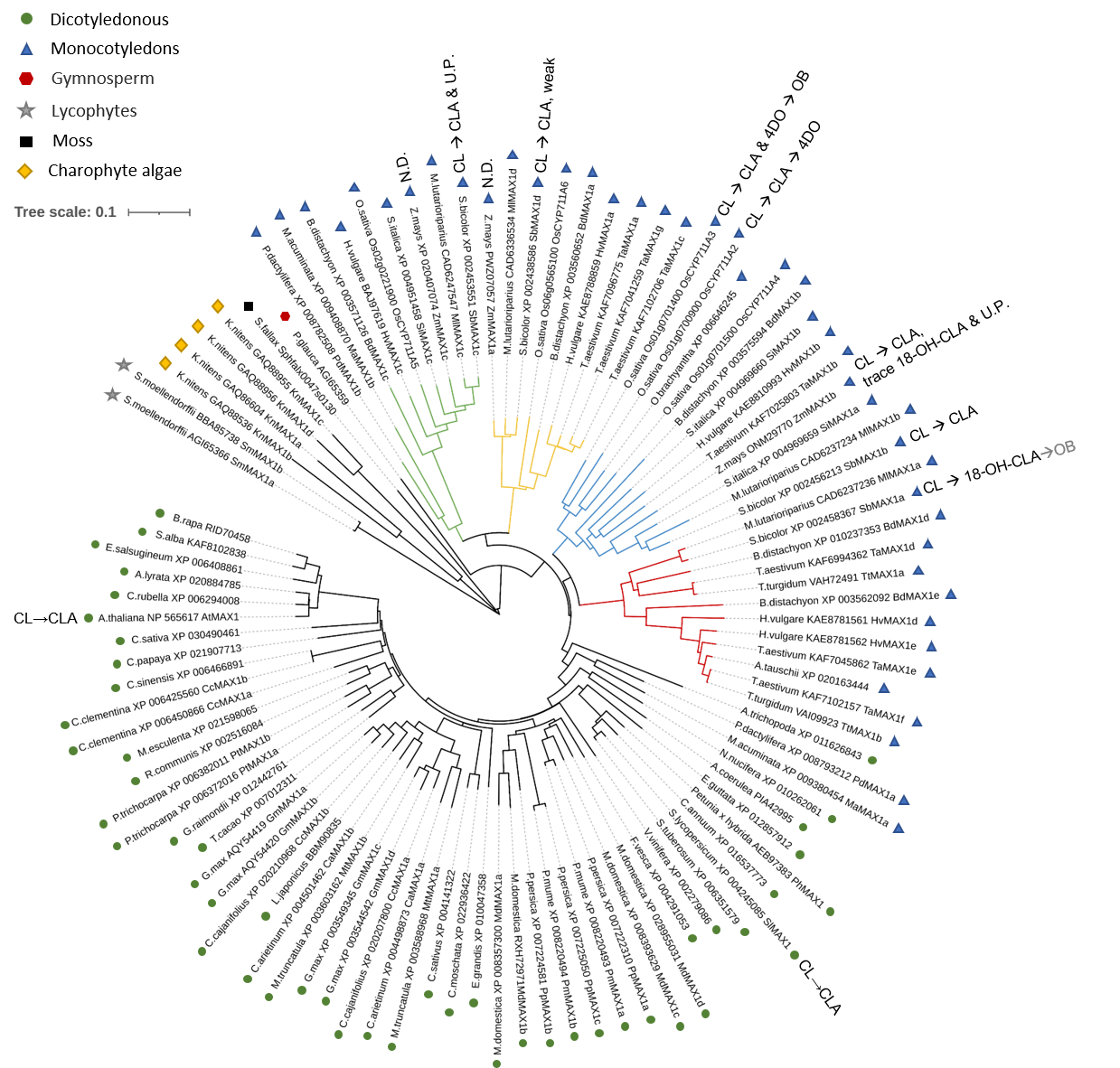
**

**Supplementary Figure 1.** Phylogenetic tree of MAX1 homologs. The phylogenetic tree was constructed by MEGA X program with neighbor joining method based on amino acid sequence (80% partial deletion, 1000 bootstraps, p-distance mode). This analysis involved 102 CYP genes (**Supplementary** **Table 6**) that belong to CYP711A clan. CYP711A from monocotyledons can be divided into four groups, which are highlighted in green, yellow, blue, and red, respectively. MAX1 analogs of confirmed functions were annotated, which can also be found in **Supplementary** **Table 1**.

**
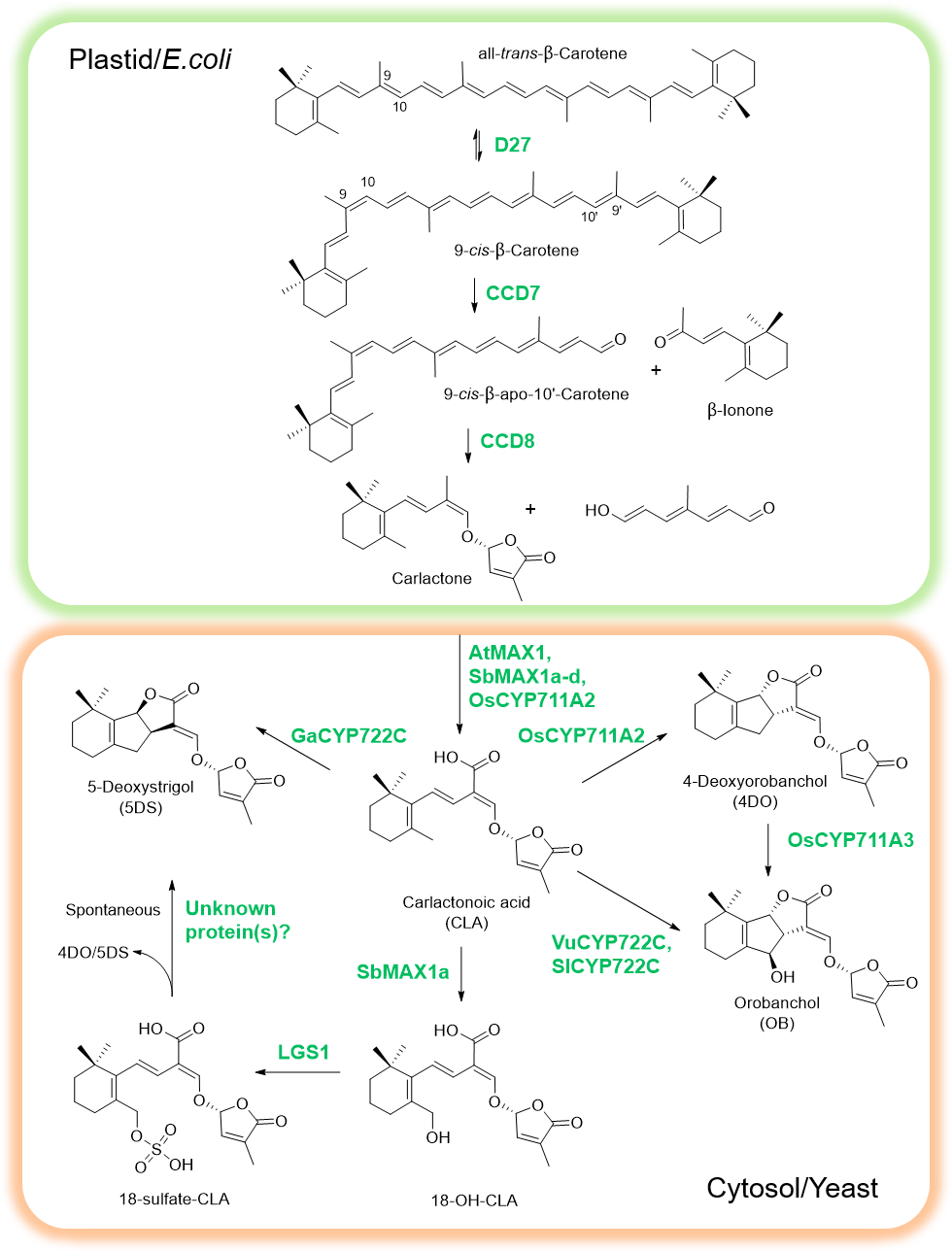
**

**Supplementary Figure 2**. Illustration of SL-producing consortium. D27, DWARF27, a [2Fe-2S]-containing isomerase; CCD7, carotenoid cleavage dioxygenase 7; CCD8, carotenoid cleavage dioxygenase 8; MAX1, MORE AXILLARY GROWTH 1, belong to CYP711A1 subfamily; CYP711A2, cytochrome P450 711A2 subfamily; CYP711A3, cytochrome P450 711A3 subfamily; CYP722C, cytochrome P450 722C subfamily; At, *Arabidopsis thaliana*; Os, *Oryza sativa*; Ga, *Gossypium arboreum*; Vu, *Vigna unguiculata*; Sl, *Solanum lycopersicum*; Sb, *Sorghum bicolor*.

**
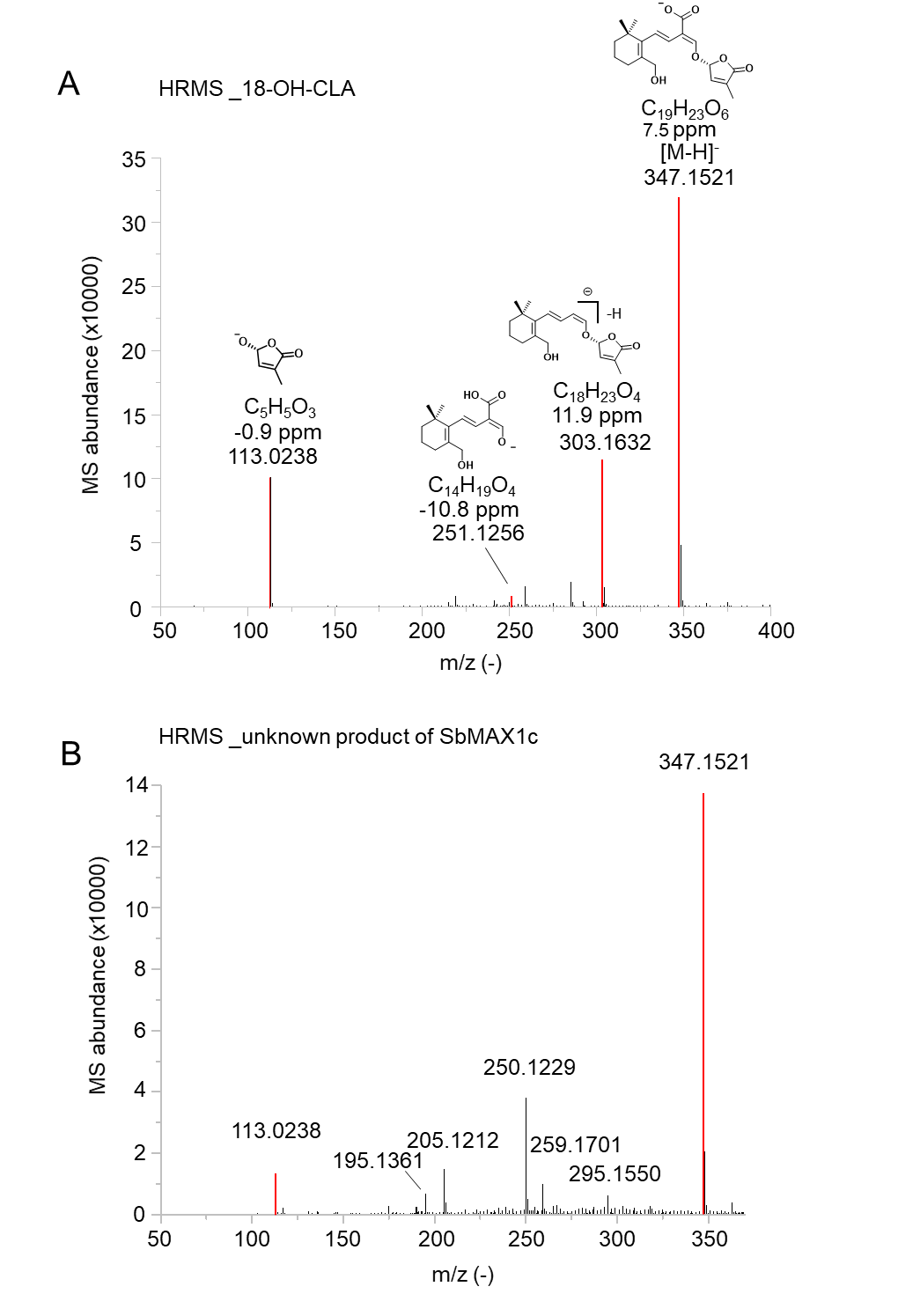
**

**Supplementary Figure 3.** HRMS analysis of 18-hydroxy-CLA and the unknown product synthesized by SbMAX1c in *E. coli-S. cerevisiae* co-culture. (**A**) HRMS of 18-hydroxy-CLA produced by CL-producing consortia **ECL/YSL2a** (**Supplementary** **Table 3**). The main fragment ions at m/z 113, 251 and 303 was consistent with the data in literature [1]. (**B**) HRMS of the unknown compound produced by CL-producing consortia **ECL**/**YSL2c** (**Supplementary** **Table 3**).


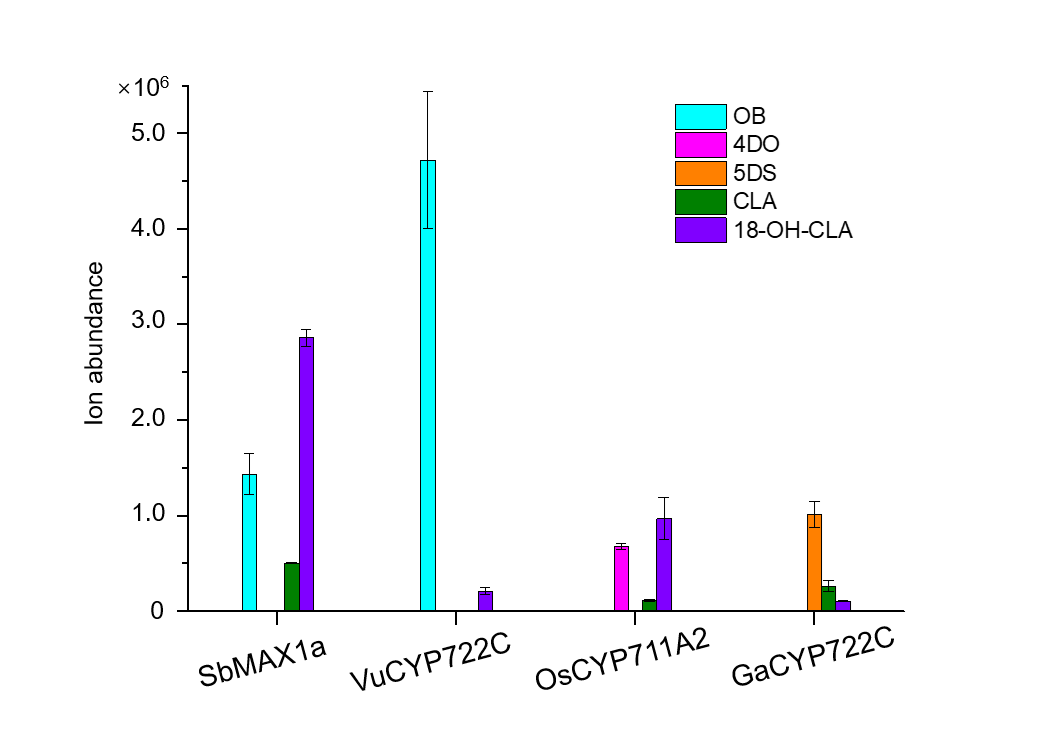


**Supplementary Figure 4.** Comparison of 18-hydroxy-CLA and OB synthesis by SbMAX1a, OsCYP711A2, VuCYP722C, and GaCYP722C in microbial consortia **ECL**/**YSL2a**, **ECL**/**YSL3**, **ECL**/**YSL4**, **ECL**/**YSL5**, respectively (**Supplementary** **Table 3**). The amounts of different SLs and intermediates was estimated using the area of the corresponding peaks of the extract ion chromatogram (EIC) at m/z^+^=347.1 (OB), m/z^+^=331.1 (4DO and 5DS), m/z^-^=331.1 (CLA), and m/z^-^=347.1 (18-hydroxy-CLA). The error bars represent the s.d. of the replicates.

**
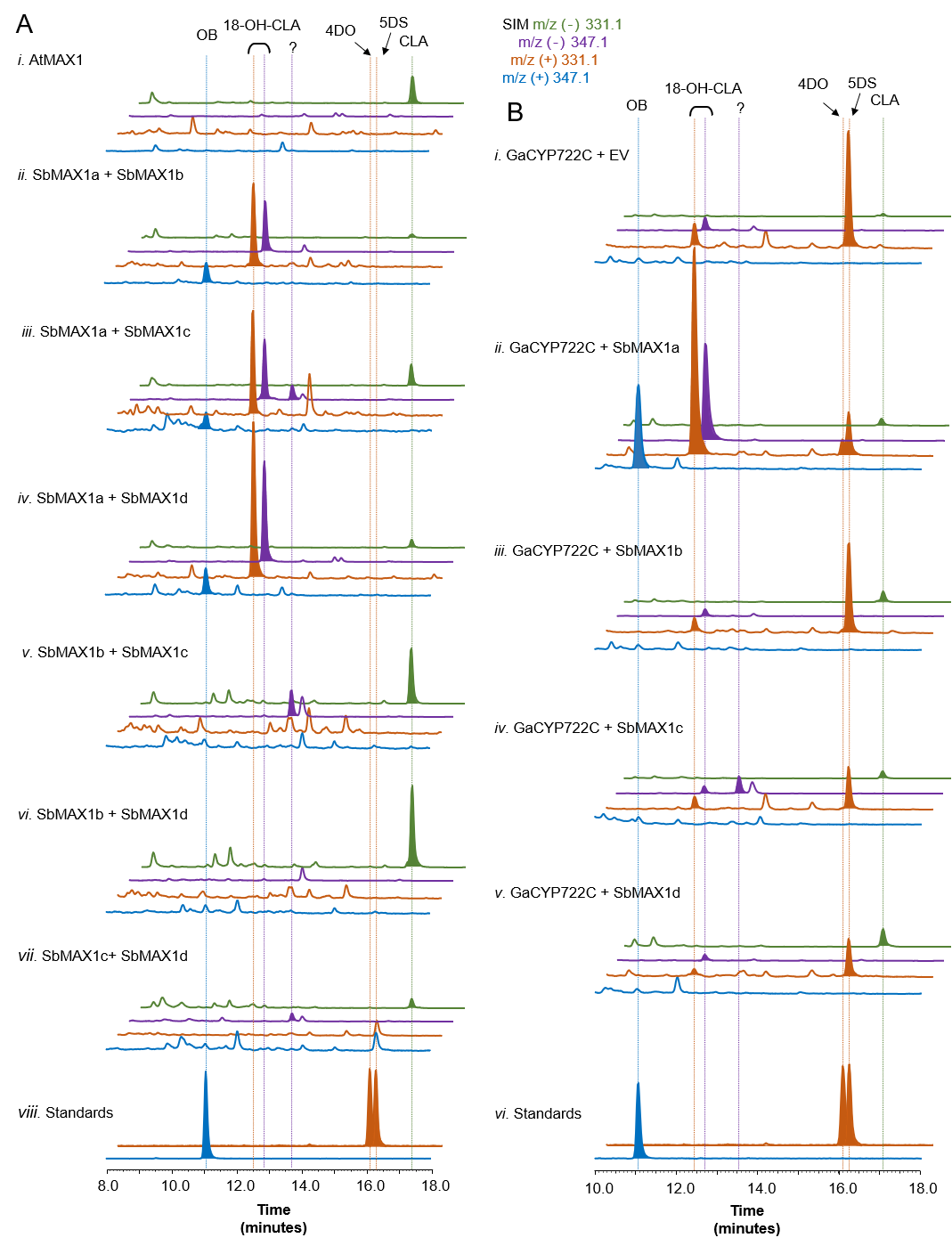
**

**Supplementary Figure 5.** LC-MS analysis of metabolites produced by consortia expressing SbMAX1a-d. (**A**) SIM EIC at m/z^-^=331.1 (green), 347.1 (purple), and m/z^+^=331.1 (orange), 347.1 (blue) of **ECL** (**Supplementary** **Table 3**) cocultured with ATR1-expressing yeast expressing i) AtMAX1 **(ECL**/**YSL1**, **Supplementary** **Table 3**); ii) SbMAX1a and SbMAX1b; iii) SbMAX1a and SbMAX1c; iv) SbMAX1a and SbMAX1d; v) SbMAX1b and SbMAX1c; vi) SbMAX1b and SbMAX1d; vii) SbMAX1c and SbMAX1d (**ECL**/**YSL6a-f**, **Supplementary** **Table 3**), and viii) standards of OB, 4DO and 5DS. (**B**) SIM EIC at m/z^-^=331.1 (green), 347.1 (purple), and m/z^+^=331.1 (orange), 347.1 (blue) of **ECL** cocultured with yeast expressing ATR1, AtMAX1, GaCYP722C and i) empty vector (EV, **ECL**/**YSL7N**, **Supplementary** **Table 3**), ii) SbMAX1a; iii) SbMAX1b; iv) SbMAX1c; v) SbMAX1d (**ECL**/**YSL7a-d**, **Supplementary** **Table 3**), and vi) standards of OB, 4DO and 5DS.


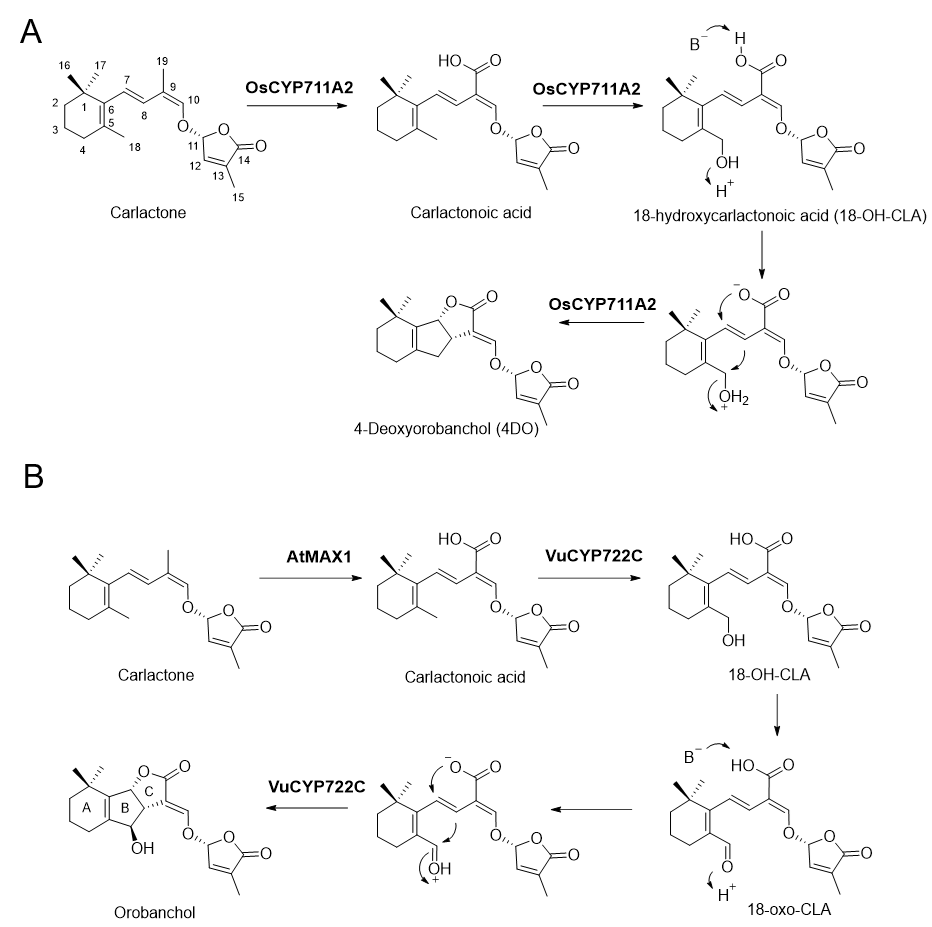


**Supplementary Figure 6.** Proposed enzymic mechanism for the synthesis of 4DO, 5DS and OB by CYP711A and CYP722C. (**A**) The hydroxylation of CLA at C-18 position to form 18-hydroxy-CLA is essential for the formation of the BC ring. 18-hydroxy-CLA is likely the precursor of canonical SL, such as 4DO, 5DS and OB. To synthesize 4DO, a proton is likely transferred to the C-18 hydroxyl group, turning it into a leaving group, which then initiates the intramolecular nucleophilic substitution reaction accompanied by the elimination of water. The synthesis of 4DO and 5DS maybe very similar, except that the stereoselectivity of BC ring formation is different, and the formation of 5DS is catalyzed by GaCYP722C. (**B**) For OB biosynthesis, the 18-position hydroxyl group of CLA can be further oxidized to aldehyde (a reactive intermediate, 18-oxo-CLA) by VuCYP722C, which triggered the BC-ring closure. A nucleophilic addition-based cyclization strategy is used here.

**
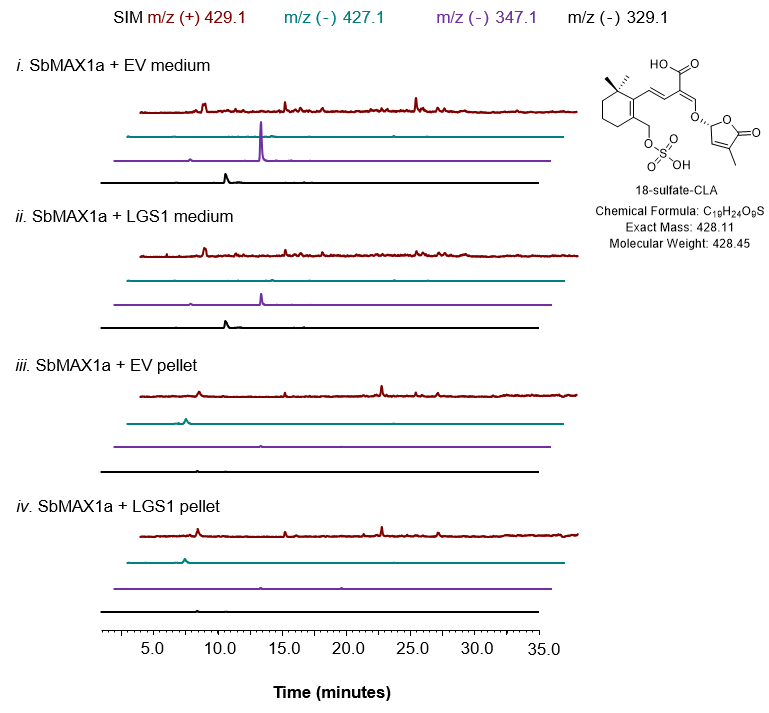
**

**Supplementary Figure 7.** 18-sulfate-CLA was not detected from either the pellets or the medium of the microbial consortia. SIM-EIC using 18-sulfate-CLA's characteristic m/z^+^ signal (MW=428.45, [C_19_H_24_O_9_S+H]^+^=[C_19_H_25_O_9_S]^+^=429.1), m/z^-^ signal ([C_19_H_24_O_9_S-H]^-^=[C_19_H_23_O_9_S]^-^=427.1, [C_19_H_24_O_9_S-HSO_3_]^-^=[C_19_H_23_O_6_]^-^=347.1, [C_19_H_24_O_9_S-H_3_SO_4_]^-^=[C_19_H_21_O_5_]^-^=329.1) of **ECL** (**Supplementary** **Table 3**) cocultured with yeast expressing ATR1, SbMAX1a and i) EV (medium extracts, **ECL**/**YSL8N**, **Supplementary** **Table 3**); ii) LGS1 (medium extracts, **ECL**/**YSL8a**, **Supplementary** **Table 3**); iii) EV (cell pellet extracts); iv) LGS1 (cell pellet extracts)

**Supplementary Figure 8.** Proposed enzymic mechanism for the synthesis of 5DS and 4DO by SbMAX1a and LGS1. SbMAX1a can directly convert CL into 18-hydroxy-CLA. Then sulfotransferase LGS1 likely catalyze the transfer of sulfate from the cofactor PAPS to the C18 hydroxyl of 18-hydroxy-CLA to form the putative unstable intermediate 18-sulfate-CLA. The sulfonate group may function as a relatively easy leaving group, and under non-enzymatic conditions, the adjacent formed carboxylate ion trigger the S_N_2′ type intramolecular nucleophilic attack and close the BC ring in a non-stereoselective manner, thereby generating approximately equal amounts of 4DO and 5DS, accompanied by the elimination of sulfate. Although the nucleophilic attack step can be carried out spontaneously as illustrated in the LGS1 assay, likely in sorghum there are additional proteins components working together with LGS1 to ensure the stereoselective conversion towards the synthesis of 5DS in sorghum.


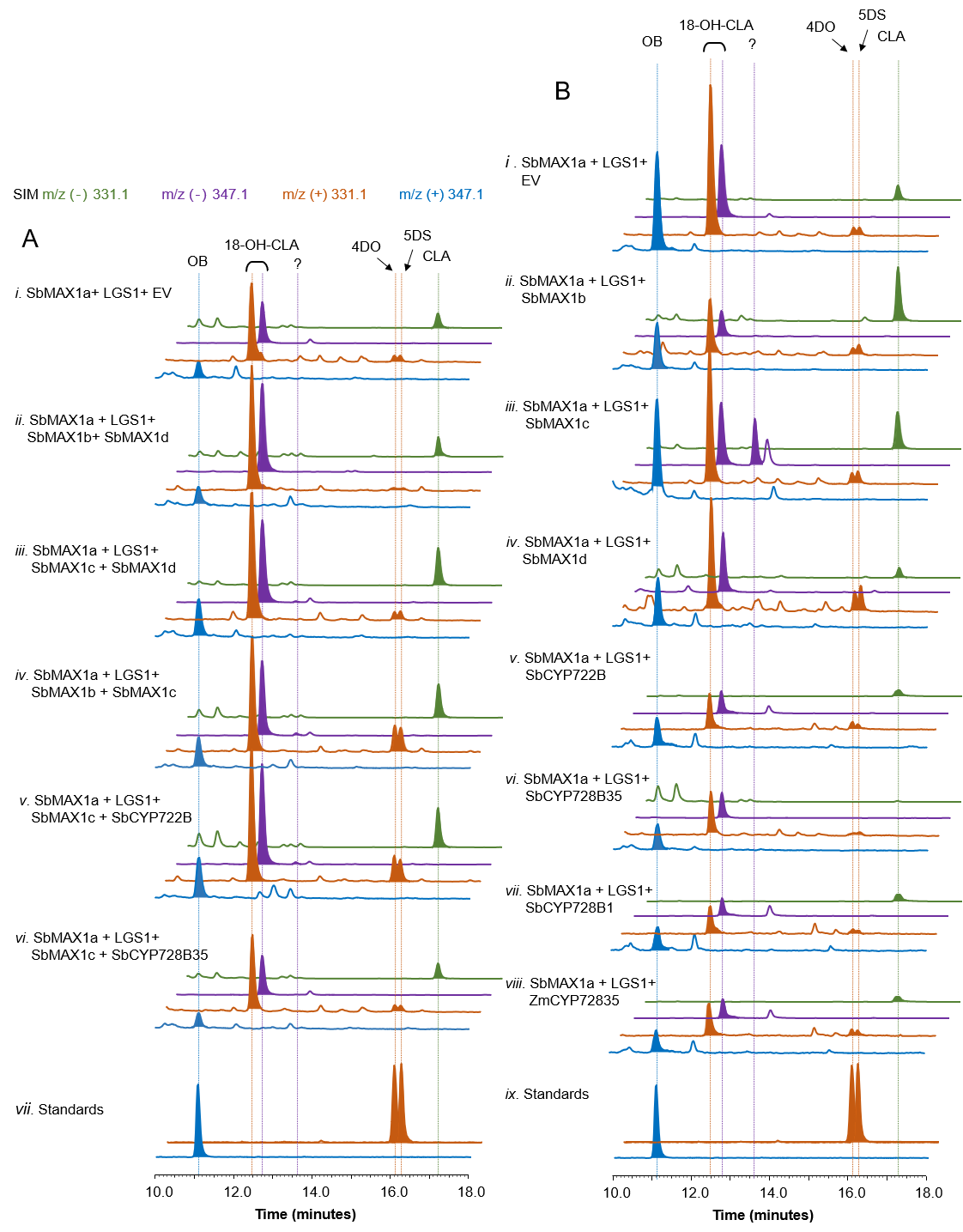


**Supplementary Figure 9.** The enzymes after LGS1 and catalyze the exclusive conversion of 5DS is missing. (**A**) SIM EIC at m/z^-^=331.1 (green), 347.1 (purple), and m/z^+^=331.1 (orange), 347.1 (blue) of **ECL** (**Supplementary** **Table 3**) cocultured with yeast strain SYL89 expressing SbMAX1a, LGS1 and i) EV (**YSL10N**, **Supplementary** **Table 3**) ; ii) SbMAX1b and SbMAX1d; iii) SbMAX1c and SbMAX1d; iv) SbMAX1b and SbMAX1c; v) SbMAX1c and SbCYP722B; vi) SbMAX1c and SbCYP728B35 (**YSL10a-e**, **Supplementary** **Table 3**); and vii) standards of OB, 4DO and 5DS. (**B**) SIM EIC at m/z^-^=331.1 (green), 347.1 (purple), and m/z^+^=331.1 (orange), 347.1 (blue) of **ECL** cocultured with yeast expressing ATR1, SbMAX1a, LGS1 and i) EV (**YSL9N**, **Supplementary** **Table 3**), ii) SbMAX1b; iii) SbMAX1c; iv) SbMAX1d; v) SbCYP722B; vi) SbCYP728B35; vii) SbCYP728B1; viii) ZmCYP728B35 (**YSL9a-g**, **Supplementary** **Table 3**) and ix) standards of OB, 4DO and 5DS.

**Supplementary Figure 10.** Full-length multiple sequence alignment of LGS1 with other SOTs. LGS1 was aligned with SOTs from plants, bacteria, and fungi using ClustalW (<https://www.genome.jp/tools-bin/clustalw>). The conserved PSB (TYPKSGT), PB (YxxRNxxDxxVS) and GxxGxxK/R motifs proposed to be critical to PAPS binding are highlighted with green box [2]. The mutated amino acid residues are marked with a red asterisk. The GenBank accession numbers of proteins analyzed are listed in **Supplementary** **Table 7**.


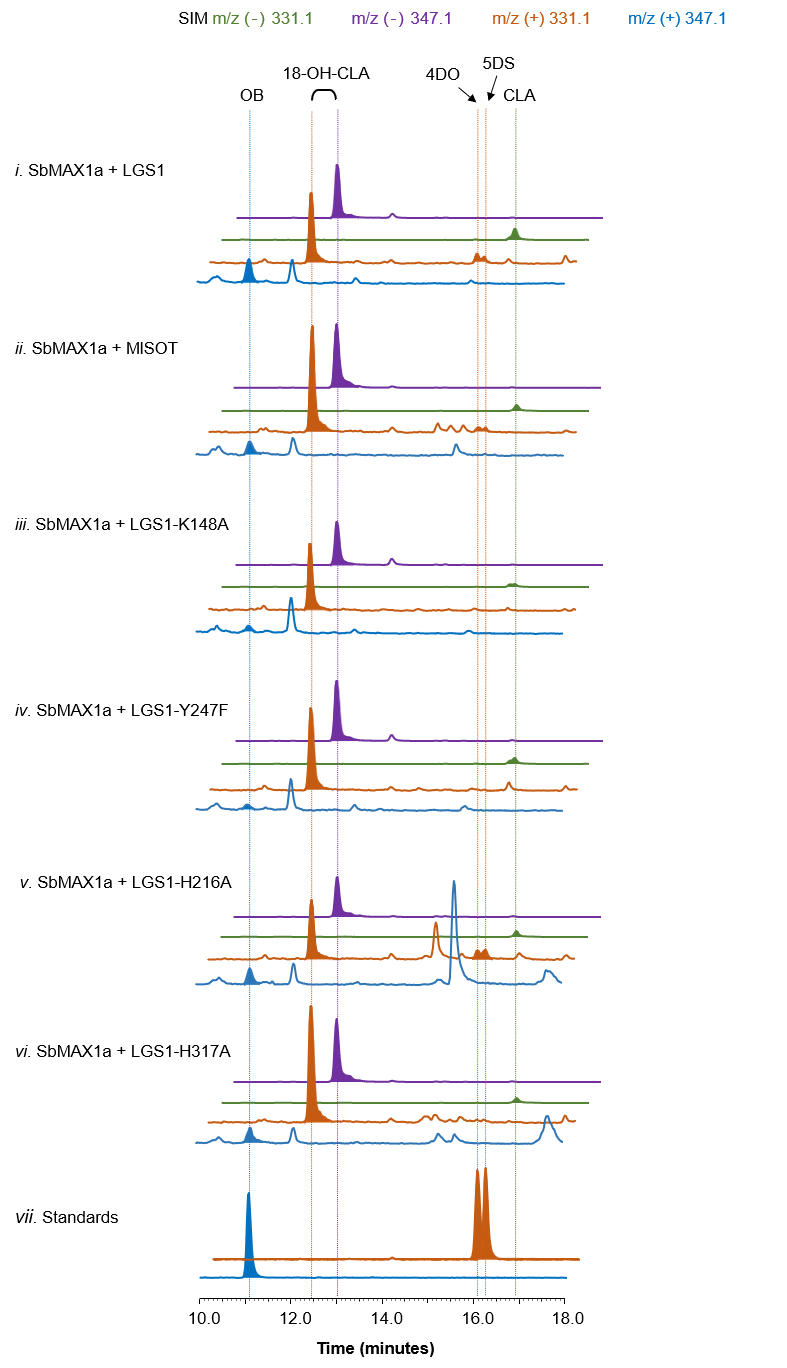


**Supplementary Figure 11.** LC-MS analysis of LGS1 mutants and MlSOT. SIM EIC at m/z^-^=331.1 (green), 347.1 (purple), and m/z^+^=331.1 (orange), 347.1 (blue) of **ECL** (**Supplementary** **Table 3**) cocultured with yeast expressing ATR1, SbMAX1a and i) LGS1 (**YSL8a**) ; ii) MlSOT (**YSL8e**); iii) LGS1^K148A^ (**YSL8g**); iv) LGS1^Y247F^ (**YSL8h**); v) LGS1^H216A^ (**YSL8f**); vi) LGS1^H317A^ (**YSL8i**); and vii) standards of OB, 4DO and 5DS. Strain information are detailed in **Supplementary** **Table 3**.


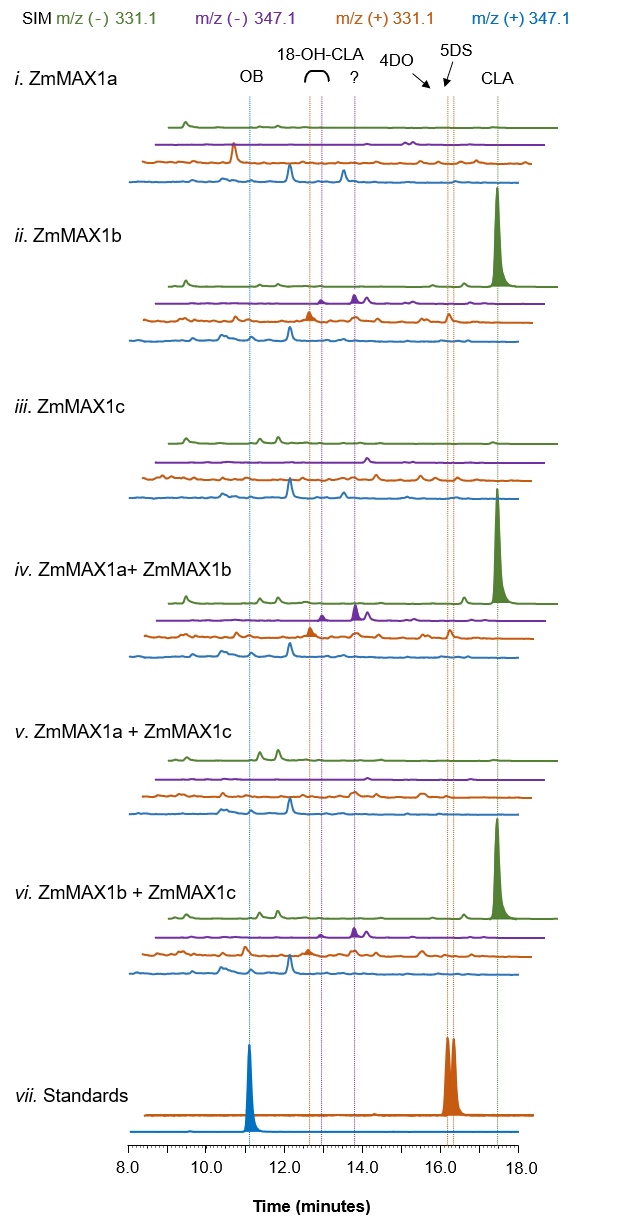


**Supplementary Figure 12.** Functional characterization of MAX1 analogs from maize. SIM EIC at m/z^-^=331.1 (green), 347.1 (purple), and m/z^+^=331.1 (orange), 347.1 (blue) of **ECL** (**Supplementary** **Table 3**) cocultured with yeast expressing ATR1 and i) ZmMAX1a; ii) ZmMAX1b; iii) ZmMAX1c; iv) ZmMAX1a and ZmMAX1b; v) ZmMAX1a and ZmMAX1c; vi) ZmMAX1b and ZmMAX1c (**YSL11a-f**, **Supplementary** **Table 3**); and vii) standards of OB, 4DO and 5DS.

**Supplementary Table 1.** CYPs used in this study and summary of results. The accession numbers of amino acid sequences of sorghum genes were extracted from Phytozome database (*Sorghum bicolor v3.1.1*; https://phytozome-next.jgi.doe.gov/), the others are from NCBI.

| **Gene** | **Species** | **Notation** | **Experimental results/product** | **Ref** |
| --- | --- | --- | --- | --- |
| *AtMAX1*  (AK316903) | *Arabidopsis thaliana* | CL 🡪 CLA | CLA | [3; 4] |
| *SbMAX1a*  (Sobic.003G269500 | *Sorghum bicolor* | CL 🡪 18-OH-CLA | CL 🡪 18-OH-CLA🡪OB | [5; 6] |
| *SbMAX1b*  (Sobic.003G269600) | *Sorghum bicolor* | CL 🡪 CLA | CL 🡪 CLA | [5; 6] |
| *SbMAX1c*  (Sobic.004G095500) | *Sorghum bicolor* | CL 🡪 CLA | CL 🡪 CLA & U.P. | [5; 6] |
| *SbMAX1d*  (Sobic.010G170400) | *Sorghum bicolor* | CL 🡪 CLA | CL 🡪 CLA, weak | [5; 6] |
| *ZmMAX1a*  (PWZ07057) | *Sorghum bicolor* | CL 🡪 CLA, weak | N.D. | [3] |
| *ZmMAX1b*  (ONM29770) | *Sorghum bicolor* | CL 🡪 CLA & 4DO 🡪 OB | CL 🡪 CLA,  trace 18-OH-CLA & U.P. | [3] |
| *ZmMAX1c*  (XP_020407074) | *Sorghum bicolor* | CL 🡪 CLA, weak | N.D. | [3] |
| *SbCYP722B*  (Sobic.009G000700) | *Sorghum bicolor* | N.D. | N.D. | [6] |
| *SbCYP728B35* (Sobic.008G122800) | *Sorghum bicolor* |  | N.D. | [5] |
| *SbCYP728B1* (Sobic.002G336100) | *Sorghum bicolor* |  | N.D. | [5] |
| *ZmCYP728B35*  (NP_001148166) | *Zea mays* |  | N.D. |  |

U.P. Unknown product; N.D. No detected; ZmMAX1a variation used in this study (PWZ07057) is nine amino acids (ASDTTMQRH) longer than ZmMAX1a (FJ957947) reported before [3]; ZmMAX1c variation (XP_020407074) used here is one amino acid (Q) longer than ZmMAX1c (NP_001145812) reported before [3].

**Supplementary Table 2.** Plasmids used in the study.

| **Plasmids** | **Description** | **Reference/ Source** |
| --- | --- | --- |
| pAC-BETAipi | Contains ctrE, crtB, crtI, crtY, and idi genes of *Erwinia herbicola* (Pantoea agglomerans) Eho10 and thereby produces beta-carotene in Escherichia coli; Derived from pACYCDuet-1, Replicon P15A (pACYC184); Resistance, Chloramphenicol; | Addgene  #53277 [7] |
| pYL726 (pCDFDuet-trAtCCD7-OsD27) | pCDFDuet-1 carrying D27 from *Oryza sativa* and N-terminus 31 amino-acid truncated CCD7 from *Arabidopsis* | [8] |
| pYL735 (pET21a-trAtCCD8) | pET21a carrying N-terminus 56 amino-acid truncated CCD8 from *Arabidopsis* | [8] |
| pAG414GPD-ccdB | *Centromeric* TRP, attR1-P_GPD_-*ccdB*-T_CYC1-_attR2 | [9] |
| pAG415GPD-ccdB | *Centromeric* LEU*,* attR1-P_GPD_-*ccdB*-T_CYC1-_attR2 | [9] |
| pAG416GPD-ccdB | *Centromeric* URA*,* attR1-P_GPD_-*ccdB*-T_CYC1-_attR2 | [9] |
| pYL573 | *Centromeric* HIS, P_TEF1-_*ATR1*-T_CYC1_ | [10] |
| pYL759 | *Centromeric* LEU*,* P_PGK1_-*AtMAX1*-T_pho5_ | [8] |
| pYL777 | *Centromeric* TRP, P_GPD_-*GaCYP722C* -T_CYC1_ | [8] |
| pYL843 | *Centromeric* URA*,* P_GPD_-*LGS1*-T_CYC1_ | This study |
| pYL885 | *Centromeric* TRP, P_GPD_-*SbMAX1b*-T_CYC1_ | This study |
| pYL891 | *Centromeric* LEU*,* P_GPD_-*SbMAX1a*-T_CYC1_ | This study |
| pYL892 | *Centromeric* URA, P_GPD_-*SbMAX1d*-T_CYC1_ | This study |
| pYL893 | *Centromeric* URA, P_GPD_-*SbMAX1c*-T_CYC1_ | This study |
| pYL896 | *Centromeric* URA*,* P_GPD_-*ZmSOT*-T_CYC1_ | This study |
| pYL1051 | *Centromeric* URA*,* P_GPD_-*TaSOT*-T_CYC1_ | This study |
| pYL1052 | *Centromeric* URA*,* P_GPD_-*ZmMAX1b*-T_CYC1_ | This study |
| pYL1053 | *Centromeric* URA*,* P_GPD_-*LGS1-2*-T_CYC1_ | This study |
| pYL1061 | *Centromeric* URA*,* P_GPD_-*SbMAX1b*-T_CYC1_ | This study |
| pYL1063 | *Centromeric* TRP, P_GPD_-*SbCYP722B*-T_CYC1_ | This study |
| pYL1079 | *Centromeric* LEU*,* P_GPD_- *ZmMAX1a*-T_CYC1_ | This study |
| pYL1080 | *Centromeric* URA, P_GPD_-*SbCYP728B35*-T_CYC1_ | This study |
| pYL1084 | *Centromeric* LEU*,* P_GPD_-*SbMAX1d*-T_CYC1_ | This study |
| pYL1085 | *Centromeric* TRP, P_GPD_-*ZmMAX1c*-T_CYC1_ | This study |
| pYL1086 | *Centromeric* LEU*,* P_GPD_-*ZmMAX1c*-T_CYC1_ | This study |
| pYL1087 | *Centromeric* LEU*,* P_GPD_-*ZmMAX1b*-T_CYC1_ | This study |
| pYL1088 | *Centromeric* URA*,* P_GPD_-*SbMAX1a*-T_CYC1_ | This study |
| pYL1090 | *Centromeric* TRP, P_GPD_-*LGS1*-T_CYC1_ | This study |
| pYL1092 | *Centromeric* TRP, P_GPD_-*SbMAX1c*-T_CYC1_ | This study |
| pYL1095 | *Centromeric* HIS*,* P_GPD_-*LGS1*-T_CYC1_ | This study |
| pYL1097 | *Centromeric* URA, P_GPD_-Zm*CYP728B35*-T_CYC1_ | This study |
| pYL1102 | *Centromeric* URA, P_GPD_-*SbCYP728B1*-T_CYC1_ | This study |
| pYL1185 | *Centromeric* URA, P_GPD_-*LGS1H216A mutant*-T_CYC1_ | This study |
| pYL1192 | *Centromeric* URA, P_GPD_-*MlSOT*-T_CYC1_ | This study |
| pYL1243 | *Centromeric* URA, P_GPD_-*LGS1Y247F mutant*-T_CYC1_ | This study |
| pYL1246 | *Centromeric* URA, P_GPD_-*LGS1K148A mutant*-T_CYC1_ | This study |
| pYL1247 | *Centromeric* URA, P_GPD_-*LGS1H317A mutant*-T_CYC1_ | This study |

*P, promoter; T, terminator*

**Supplementary Table 3.** Strains used in the study.

| **Yeast Strain** | **Description** | **Genotype** | **Function** | **Reference** |
| --- | --- | --- | --- | --- |
| CEN.PK2-1D |  | *MATα; his3D1; leu2-3_112; ura3-52; trp1-289; MAL2-8c; SUC2* | Wild type yeast strain | [11] |
| SYL89 |  | CEN.PK2-1D, Leu2∆::PTEF1-ATR1-TCYC1 | Yeast strain expressing the *A. thaliana* P450 reductase 1 | This study |
| YSL1 | CEN.PK2-1D carrying pYL573 and pYL759 | Centromeric HIS, *P_TEF1-_ATR1-T_CYC1_*,  Centromeric URA, P_GPD_-*AtMAX1*-T_CYC1_ | CLA production | This study |
| YSL2a | CEN.PK2-1D carrying pYL573 and pYL891 | Centromeric HIS, *P_TEF1-_ATR1-T_CYC1_*,  Centromeric LEU, P_GPD_-*SbMAX1a*-T_CYC1_ | OB and 18-OH-CLA production | This study |
| YSL2b | CEN.PK2-1D carrying  pYL573, pYL885 and pAG416GPD-ccdB | Centromeric HIS, *P_TEF1-_ATR1-T_CYC1_*,  Centromeric TRP, P_GPD_-*SbMAX1b*-T_CYC1_,  Centromeric URA, P_GPD_-*ccdB*-T_CYC1_ | CLA production | This study |
| YSL2c | CEN.PK2-1D carrying pYL573 and pYL893 | Centromeric HIS, *P_TEF1-_ATR1-T_CYC1_*,  Centromeric URA, P_GPD_-*SbMAX1c*-T_CYC1_ | CLA and unknown production | This study |
| YSL2d | CEN.PK2-1D carrying pYL573 and pYL892 | Centromeric HIS, *P_TEF1-_ATR1-T_CYC1_*,  Centromeric URA, P_GPD_-*SbMAX1d*-T_CYC1_ | CLA production | This study |
| YSL3 | CEN.PK2-1D carrying pYL573 and pYL770 | Centromeric HIS, *P_TEF1-_ATR1-T_CYC1_*,  Centromeric URA, *P_PGK1_-OsCYP711A2-T_PHO5_* | 4DO production | [8] |
| YSL4 | CEN.PK2-1D carrying pYL573, pYL759, and pYL1070 | Centromeric HIS, *P_TEF1-_ATR1-T_CYC1_*,  Centromeric LEU, *P_PGK1_-AtMAX1-T_PHO5_*,  Centromeric TRP, *P_GPD_-VuCYP722C-T_CYC1_* | OB production | [8] |
| YSL5 | CEN.PK2-1D carrying pYL573, pYL759, and pYL777 | Centromeric HIS, *P_TEF1-_ATR1-T_CYC1_*,  Centromeric LEU, *P_PGK1_-AtMAX1-T_PHO5_*,  Centromeric TRP, *P_GPD_-GaCYP722C-T_CYC1_* | 5DS production | [8] |
| YSL6a | CEN.PK2-1D carrying pYL573, pYL891 and pYL885 | Centromeric HIS, *P_TEF1-_ATR1-T_CYC1_*,  Centromeric LEU*,* P_GPD_-*SbMAX1a*-T_CYC1_,  Centromeric TRP, P_GPD_-*SbMAX1b*-T_CYC1_, | OB and 18-OH-CLA production | This study |
| YSL6b | CEN.PK2-1D carrying pYL573, pYL891 and pYL892 | Centromeric HIS, *P_TEF1-_ATR1-T_CYC1_*,  Centromeric LEU, P_GPD_-*SbMAX1a*-T_CYC1_,  Centromeric URA, P_GPD_-*SbMAX1d*-T_CYC1_ | OB and 18-OH-CLA production | This study |
| YSL6c | CEN.PK2-1D carrying pYL573, pYL891 and pYL893 | Centromeric HIS, *P_TEF1-_ATR1-T_CYC1_*,  Centromeric LEU, P_GPD_-*SbMAX1a*-T_CYC1_,  Centromeric URA, P_GPD_-*SbMAX1c*-T_CYC1_ | OB ,18-OH-CLA and unknown production | This study |
| YSL6d | CEN.PK2-1D carrying pYL573, pYL885 and pYL892 | Centromeric HIS, *P_TEF1-_ATR1-T_CYC1_*, Centromeric TRP, P_GPD_-*SbMAX1b*-T_CYC1_,  Centromeric URA, P_GPD_-*SbMAX1d*-T_CYC1_ | CLA production | This study |
| YSL6e | CEN.PK2-1D carrying pYL573, pYL885 and pYL893 | Centromeric HIS, *P_TEF1-_ATR1-T_CYC1_*, Centromeric TRP, P_GPD_-*SbMAX1b*-T_CYC1_,  Centromeric URA, P_GPD_-*SbMAX1c*-T_CYC1_ | CLA and unknown production | This study |
| YSL6f | CEN.PK2-1D carrying pYL573, pYL1084 and pYL893 | Centromeric HIS, *P_TEF1-_ATR1-T_CYC1_*, Centromeric LEU, P_GPD_-*SbMAX1d*-T_CYC1_,  Centromeric URA, P_GPD_-*SbMAX1c*-T_CYC1_ | Unknown product | This study |
| YSL7N | CEN.PK2-1D carrying pYL573, pYL759, pYL777 and pAG416GPD-ccdB | Centromeric HIS, *P_TEF1-_ATR1-T_CYC1_*,  Centromeric LEU, *P_PGK1_-AtMAX1-T_PHO5_*,  Centromeric TRP, *P_GPD_-GaCYP722C-T_CYC1_*  Centromeric URA*,* P_GPD_-*ccdB*-T_CYC1_ | Negative control for  YSL-7 | This study |
| YSL7a | EN.PK2-1D carrying pYL573, pYL759, pYL777 and pYL1088 | Centromeric HIS, *P_TEF1-_ATR1-T_CYC1_*,  Centromeric LEU, *P_PGK1_-AtMAX1-T_PHO5_*,  Centromeric TRP, *P_GPD_-GaCYP722C-T_CYC1_*  Centromeric URA*,* P_GPD_-*SbMAX1a*-T_CYC1_ | 5DS production, no downstream products detected | This study |
| YSL7b | CEN.PK2-1D carrying pYL573, pYL759, pYL777 and pYL1061 | Centromeric HIS, *P_TEF1-_ATR1-T_CYC1_*,  Centromeric LEU, *P_PGK1_-AtMAX1-T_PHO5_*,  Centromeric TRP, *P_GPD_-GaCYP722C-T_CYC1_*  Centromeric URA*,* P_GPD_-*SbMAX1b*-T_CYC1_ | 5DS production, no downstream products detected | This study |
| YSL7c | CEN.PK2-1D carrying pYL573, pYL759, pYL777 and pYL893 | Centromeric HIS, *P_TEF1-_ATR1-T_CYC1_*,  Centromeric LEU, *P_PGK1_-AtMAX1-T_PHO5_*,  Centromeric TRP, *P_GPD_-GaCYP722C-T_CYC1_*  Centromeric URA*,* P_GPD_-*SbMAX1c*-T_CYC1_ | 5DS production, no downstream products detected | This study |
| YSL7d | CEN.PK2-1D carrying pYL573, pYL759, pYL777 and pYL892 | Centromeric HIS, *P_TEF1-_ATR1-T_CYC1_*,  Centromeric LEU, *P_PGK1_-AtMAX1-T_PHO5_*,  Centromeric TRP, *P_GPD_-GaCYP722C-T_CYC1_*  Centromeric URA*,* P_GPD_-*SbMAX1d*-T_CYC1_ | 5DS production, no downstream products detected | This study |
| YSL8N | CEN.PK2-1D carrying pYL573, pYL891 and pAG416GPD-ccdB | Centromeric HIS, *P_TEF1-_ATR1-T_CYC1_*,  Centromeric LEU, P_GPD_-*SbMAX1a*-T_CYC1_,  Centromeric URA P_GPD_-*ccdB*-T_CYC1_ | Negative control for  YSL-8 | This study |
| YSL8a | CEN.PK2-1D carrying pYL573, pYL891 and pYL843 | Centromeric HIS, *P_TEF1-_ATR1-T_CYC1_*,  Centromeric LEU, P_GPD_-*SbMAX1a*-T_CYC1_,  *Centromeric* URA*,* P_GPD_-*LGS1*-T_CYC1_ | LGS1 is involved in the conversion of 18-OH-CLA to 4DO/5DS | This study |
| YSL8b | CEN.PK2-1D carrying pYL573, pYL891 and pYL1053 | Centromeric HIS, *P_TEF1-_ATR1-T_CYC1_*,  Centromeric LEU*,* P_GPD_-*SbMAX1a*-T_CYC1_,  *Centromeric* URA*,* P_GPD_-*LGS1-2*-T_CYC1_ | LGS1-2 is involved in the conversion of 18-OH-CLA to 4DO/5DS | This study |
| YSL8c | CEN.PK2-1D carrying pYL573, pYL891 and pYL1051 | Centromeric HIS, *P_TEF1-_ATR1-T_CYC1_*,  Centromeric LEU*,* P_GPD_-*SbMAX1a*-T_CYC1_,  *Centromeric* URA*,* P_GPD_-*TaSOT*-T_CYC1_ | Failed functional characterization of TaSOT | This study |
| YSL8d | CEN.PK2-1D carrying pYL573, pYL891 and pYL896 | Centromeric HIS, *P_TEF1-_ATR1-T_CYC1_*,  Centromeric LEU*,* P_GPD_-*SbMAX1a*-T_CYC1_,  *Centromeric* URA*,* P_GPD_-*ZmSOT*-T_CYC1_ | Failed functional characterization of ZmSOT | This study |
| YSL8e | CEN.PK2-1D carrying pYL573, pYL891 and pYL1192 | Centromeric HIS, *P_TEF1-_ATR1-T_CYC1_*,  Centromeric LEU*,* P_GPD_-*SbMAX1a*-T_CYC1_,  *Centromeric* URA*,* P_GPD_-*MlSOT*-T_CYC1_ | Trace 4DO/5DS production | This study |
| YSL8f | CEN.PK2-1D carrying pYL573, pYL891 and pYL1185 | Centromeric HIS, *P_TEF1-_ATR1-T_CYC1_*,  Centromeric LEU*,* P_GPD_-*SbMAX1a*-T_CYC1_,  *Centromeric* URA*,* P_GPD_- *LGS1H216A mutant* -T_CYC1_ | 4DO/5DS production | This study |
| YSL8g | CEN.PK2-1D carrying pYL573, pYL891 and pYL1246 | Centromeric HIS, *P_TEF1-_ATR1-T_CYC1_*,  Centromeric LEU*,* P_GPD_-*SbMAX1a*-T_CYC1_,  *Centromeric* URA*,* P_GPD_- *LGS1K148A mutant* -T_CYC1_ | No 4DO/5DS production | This study |
| YSL8h | CEN.PK2-1D carrying pYL573, pYL891 and pYL1243 | Centromeric HIS, *P_TEF1-_ATR1-T_CYC1_*,  Centromeric LEU*,* P_GPD_-*SbMAX1a*-T_CYC1_,  *Centromeric* URA*,* P_GPD_- *LGS1Y247F mutant* -T_CYC1_ | No 4DO/5DS production | This study |
| YSL8i | CEN.PK2-1D carrying pYL573, pYL891 and pYL1247 | Centromeric HIS, *P_TEF1-_ATR1-T_CYC1_*,  Centromeric LEU*,* P_GPD_-*SbMAX1a*-T_CYC1_,  *Centromeric* URA*,* P_GPD_- *LGS1H317A mutant* -T_CYC1_ | No 4DO/5DS production | This study |
| YSL9N | CEN.PK2-1D carrying pYL573, pYL891, pYL1090 and pAG416GPD-ccdB | Centromeric HIS, *P_TEF1-_ATR1-T_CYC1_*, Centromeric LEU, *P*_GPD_-*SbMAX1a*-*T*_CYC1_,  Centromeric TRP, *P_GPD_-LGS1-T_CYC1_*,  Centromeric URA*,* *P*_GPD_-*ccdB*-*T*_CYC1_ | Negative control for  YSL-9 | This study |
| YSL9a | CEN.PK2-1D carrying pYL573, pYL891 pYL1090 and pYL892 | Centromeric HIS, *P_TEF1-_ATR1-T_CYC1_*, Centromeric LEU, *P*_GPD_-*SbMAX1a*-*T*_CYC1_,  Centromeric TRP, *P_GPD_-LGS1-T_CYC1_*,  Centromeric URA*,* *P*_GPD_-*SbMAX1d*-*T*_CYC1_ | No change in 4DO/5DS ratio or new products detected | This study |
| YSL9b | CEN.PK2-1D carrying pYL573, pYL891, pYL885 and pYL843 | Centromeric HIS, *P_TEF1-_ATR1-T_CYC1_*, Centromeric LEU, *P*_GPD_-*SbMAX1a*-*T*_CYC1_,  Centromeric TRP, *P_GPD_-SbMAX1b-T_CYC1_*,  Centromeric URA*,* *P*_GPD_-*LGS1*-*T*_CYC1_ | No change in 4DO/5DS ratio or new products detected | This study |
| YSL9c | CEN.PK2-1D carrying pYL573, pYL891, pYL1090 and pYL893 | Centromeric HIS, *P_TEF1-_ATR1-T_CYC1_*, Centromeric LEU, *P*_GPD_-*SbMAX1a*-*T*_CYC1_,  Centromeric TRP, *P_GPD_-LGS1-T_CYC1_*,  Centromeric URA*,* *P*_GPD_-*SbMAX1c*-*T*_CYC1_ | No change in 4DO/5DS ratio or new products detected | This study |
| YSL9d | CEN.PK2-1D carrying pYL573, pYL891, pYL843 and pYL1063 | Centromeric HIS, *P_TEF1-_ATR1-T_CYC1_*, Centromeric LEU, *P*_GPD_-*SbMAX1a*-*T*_CYC1_,  Centromeric URA, *P_GPD_-LGS1-T_CYC1_*,  Centromeric TRP*,* *P*_GPD_-*SbCYP722B*-*T*_CYC1_ | No change in 4DO/5DS ratio or new products detected | This study |
| YSL9e | CEN.PK2-1D carrying pYL573, pYL891, pYL1090 and pYL1080 | Centromeric HIS, *P_TEF1-_ATR1-T_CYC1_*, Centromeric LEU, *P*_GPD_-*SbMAX1a*-*T*_CYC1_,  Centromeric TRP, *P_GPD_-LGS1-T_CYC1_*,  Centromeric URA*,* *P*_GPD_-*SbCYP728B35*-*T*_CYC1_ | No change in 4DO/5DS ratio or new products detected | This study |
| YSL9f | CEN.PK2-1D carrying pYL573, pYL891, pYL1090 and pYL1102 | Centromeric HIS, *P_TEF1-_ATR1-T_CYC1_*, Centromeric LEU, *P*_GPD_-*SbMAX1a*-*T*_CYC1_,  Centromeric TRP, *P_GPD_-LGS1-T_CYC1_*,  Centromeric URA*,* *P*_GPD_-*SbCYP728B1*-*T*_CYC1_ | No change in 4DO/5DS ratio or new products detected | This study |
| YSL9g | CEN.PK2-1D carrying pYL573, pYL891, pYL1090 and pYL1097 | Centromeric HIS, *P_TEF1-_ATR1-T_CYC1_*, Centromeric LEU, *P*_GPD_-*SbMAX1a*-*T*_CYC1_,  Centromeric TRP, *P_GPD_-LGS1-T_CYC1_*,  Centromeric URA*,* *P*_GPD_-*ZmCYP728B35*-*T*_CYC1_ | No change in 4DO/5DS ratio or new products detected | This study |
| YSL10N | SYL89 carrying pYL1095, pYL891,  pAG414GPD-ccdB and pAG416GPD-ccdB | Centromeric HIS, *P*_GPD_-*LGS1*-*T*_CYC1_,  Centromeric LEU, *P*_GPD_-*SbMAX1a*-*T*_CYC1_,  Centromeric TRP, *P_GPD_-ccdB-T_CYC1_*,  Centromeric URA*,* *P*_GPD_-*ccdB*-*T*_CYC1_ | Negative control for  YSL-10 | This study |
| YSL10a | SYL89 carrying pYL1095, pYL891, pYL885 and pYL892 | Centromeric HIS, *P*_GPD_-*LGS1*-*T*_CYC1_,  Centromeric LEU, *P*_GPD_-*SbMAX1a*-*T*_CYC1_,  Centromeric TRP, *P_GPD_-SbMAX1b-T_CYC1_*,  Centromeric URA*,* *P*_GPD_-*SbMAX1d*-*T*_CYC1_ | No change in 4DO/5DS ratio or new products detected | This study |
| YSL10b | SYL89 carrying pYL1095, pYL891, pYL1092 and pYL892 | Centromeric HIS, *P*_GPD_-*LGS1*-*T*_CYC1_,  Centromeric LEU, *P*_GPD_-*SbMAX1a*-*T*_CYC1_,  Centromeric TRP, *P_GPD_-SbMAX1c-T_CYC1_*,  Centromeric URA*,* *P*_GPD_-*SbMAX1d*-*T*_CYC1_ | No change in 4DO/5DS ratio or new products detected | This study |
| YSL10c | SYL89 carrying pYL1095, pYL891, pYL885 and pYL893 | Centromeric HIS, *P*_GPD_-*LGS1*-*T*_CYC1_,  Centromeric LEU, *P*_GPD_-*SbMAX1a*-*T*_CYC1_,  Centromeric TRP, *P_GPD_-SbMAX1b-T_CYC1_*,  Centromeric URA*,* *P*_GPD_-*SbMAX1c* -*T*_CYC1_ | No change in 4DO/5DS ratio or new products detected | This study |
| YSL10d | SYL89 carrying pYL1095, pYL891, pYL1063 and pYL893 | Centromeric HIS, *P*_GPD_-*LGS1*-*T*_CYC1_,  Centromeric LEU, *P*_GPD_-*SbMAX1a*-*T*_CYC1_,  Centromeric TRP, *P_GPD_-SbCYP722B-T_CYC1_*,  Centromeric URA*,* *P*_GPD_-*SbMAX1c*-*T*_CYC1_ | No change in 4DO/5DS ratio or new products detected | This study |
| YSL10e | SYL89 carrying pYL1095, pYL891, pYL1092 and pYL1080 | Centromeric HIS, *P*_GPD_-*LGS1*-*T*_CYC1_,  Centromeric LEU, *P*_GPD_-*SbMAX1a*-*T*_CYC1_,  Centromeric TRP, *P_GPD_-SbMAX1c-T_CYC1_*,  Centromeric URA*,* *P*_GPD_-*SbCYP728B35*-*T*_CYC1_ | No change in 4DO/5DS ratio or new products detected | This study |
| YSL11a | CEN.PK2-1D carrying pYL573 and pYL1079 | Centromeric HIS, *P_TEF1-_ATR1-T_CYC1_*,  *Centromeric* LEU*,* P_GPD_-*ZmMAX1a*-T_CYC1_ | Failed functional characterization of ZmMAX1a | This study |
| YSL11b | CEN.PK2-1D carrying pYL573 and pYL1087 | Centromeric HIS, *P_TEF1-_ATR1-T_CYC1_*,  *Centromeric* LEU*,* P_GPD_-*ZmMAX1b*-T_CYC1_ | CLA, trace 18-OH-CLA and unknown product | This study |
| YSL11c | CEN.PK2-1D carrying pYL573 and pYL1086 | Centromeric HIS, *P_TEF1-_ATR1-T_CYC1_*,  *Centromeric* LEU*,* P_GPD_-*ZmMAX1c*-T_CYC1_ | Failed functional characterization of ZmMAX1c | This study |
| YSL11d | CEN.PK2-1D carrying pYL573, pYL1079 and pYL1052 | Centromeric HIS, *P_TEF1-_ATR1-T_CYC1_*,  *Centromeric* LEU*,* P_GPD_-*ZmMAX1a*-T_CYC1_, *Centromeric* URA*,* P_GPD_-*ZmMAX1b*-T_CYC1_ | CLA, trace 18-OH-CLA and unknown product | This study |
| YSL11e | CEN.PK2-1D carrying pYL573, pYL1079 and pYL1085 | Centromeric HIS, *P_TEF1-_ATR1-T_CYC1_*,  *Centromeric* LEU*,* P_GPD_-*ZmMAX1a*-T_CYC1_,  *Centromeric* TRP, P_GPD_-*ZmMAX1c*-T_CYC1_ | Failed functional characterization of ZmMAX1a/ ZmMAX1c | This study |
| YSL11f | CEN.PK2-1D carrying pYL573, pYL1087 and pYL1085 | Centromeric HIS, *P_TEF1-_ATR1-T_CYC1_*, *Centromeric* LEU*,* P_GPD_-*ZmMAX1b*-T_CYC1_,  *Centromeric* TRP, P_GPD_-*ZmMAX1c*-T_CYC1_ | CLA, trace 18-OH-CLA and unknown product | This study |
| ***E. coli* Strain** | **Base Strain** | **Plasmid** | **Function** |  |
| ECL | BL21(DE3) | pAC-BETAipi;  pYL726 (pCDFDuet-trAtCCD7-OsD27);  pYL735 (pET21a-trAtCCD8) | CL production | [8] |

**Supplementary Table 4.** Sequences of genes used in this study

| **Gene** | **Sequence (5′–3′)** |
| --- | --- |
| *SbMAX1a* | ATGGGTTGGGGTGAAATTATCTCTTCCCAGTTGTTGATCGAGTCCTCTTCATCTTCTTTGCCAGCTGTTTTGTTTACTGCTGCTGCTTTGGCTGCTGGTGCATTTGCTGTTTATTTCTATATTCCATCTTGGAGAGTCAGAAGAGTTCCAGGTCCAGTTGCTTTGCCATTGGTTGGTCATTTGCCATTATTTGCTAAACATGGTCCAGGTTTGTTCAGGATGTTGGCTAAAGAATATGGTCCAATCTACAGATTCCACATGGGTAGACAACCATTGGTTATGGTTGCTGATGCTGAATTGTGTAAAGAAGTCGGCATTAAGAAGTTCAAGTCCATTCCAAACAGATCTATCCCAACTCCAATTAGAGGTTCCCCAATTCATAACAAGGGTTTGTTCTTCACCAGAGACTCTAGATGGCAATCTATGAGAAACGTTATCTTGACCATCTACCAACCATCTCATGTTGCTTCATTGATTCCAGCTATTCAACCATACGTTGAAAGAGCCGGTAGATTATTGCATCCAGGTGAAGAAATTACCTTCTCCGATTTGTCTCTGAAGTTGTTCAATGATACCATTGGTCAAGTTGCCTTCGGTGTTGATTTTGGTTTGACTAAGGATGATACAACTGCTGCTACTTCTCCAGCTGCTCAACAACAACCAGCTCATGGTGGTGCTAATGCTAATCAATCTGTTGATGATCCAGCCACCGATTTCATTAGAAAACATTTTAGAGCTACCACCAGCCTGAAGATGGATTTGTCTGGTCCATTGTCTATAGTCTTGGGTCAATTTGTTCCATTCCTGCAAGAACCAGTTAGACAGTTGATGTTGAGAGTTCCTGGTTCTGCTGATAGAAGATTGGAAGAGGCTAATTCTGATATGTCTGGTTTGTTGGACGAAATCGTTGCTGAAAGAGCTGCACAAGCTGATAGAGGTCAACAAAAGAATTTCCTGTCCGTTTTGTTGAACGCTAGAGAATCTACTGAAGCCATGAAGAAGTTGTTGACTCCAGATTATGTTTCCGCTTTGACCTACGAACATTTGTTGGCTGGTTCTGTTACTATGTCTTTCACCTTGTCATCCTTGGTTTACTTGGTTGCTATGCATCCAGAAGTCGAAGAAAAGCTGTTGAGAGAAATTGATGCTTTCGGTCCAAAAGATGTTGTTCCATCTTCTGATGACTTGGAGACTAAGTTCCCATATGTTGAACAAGTCGTCAAAGAAACCATGAGATTCTATACTGCTTCACCTTTGGTTGCAAGACAAGCTTCTGAAGATGTTGAAGTTGGTGGTTACTTGTTGCCAAAAGGTACTTGGGTTTGGTTGGCTCCAGGTGTTTTGGCAAAAGATCCTAAAGATTTTCCAGATCCAGACGTGTTTAGACCAGAAAGATTTGATCCTGAATCCGAAGAATGTAAGAGAAGGCATCCATACGCTTTTATTCCATTTGGTATTGGTCCAAGAGCCTGTATTGGTCAAAAATTCGCTATGCAGCAACTGAAATTGGTTGTCATCCACTTGTACCGTAACTACATTTTCAGACATTCCCCAAGAATGGAATTCCCATTGCAATTCCAATACTCGATCTTGGTCAACTTTAAGTACGGTGTTAAGGTGCAAGTCATCGAGAGAAAGAACTGA |
| *SbMAX1b* | ATGGAAATGGGTACTGTTTTGGGTGCTATGGAAGAGTACACTTTTACTTTTTTGGCTATGGCCGTTGGTTTCTTGGTTTTGGTTTACTTGTATGAGCCATACTGGAAGGTTAGACATGTTCCAGGTCCAGTTCCATTGCCATTGATTGGTCACTTGCATTTGTTGGCTAAACATGGTCCAGATGTTTTTCCAGTTTTGGCCAAGAAACACGGTCCAATTTTTAGATTCCATGTCGGTAGACAACCATTGATTATAGTTGCTGATGCCGAATTGTGCAAAGAAGTCGGTATTAAGAAATTCAAGTCCATGCCAAACAGGTCTTTGCCATCTCCAATTGCTAATTCCCCAATTCATAGAAAGGGTTTGTTCGCTACTAGAGACTCTAGATGGTCTGCTATGAGAAACGTTATTGTCTCTATCTACCAACCATCTCATTTGGCTGGTTTGATGCCAACTATGGAATCTTGTATTGAAAGAGCTGCTACCACCAACTTAGGTGATGGTGAAGAAGTTGTTTTCTCCAAGTTGGCTTTGTCTTTGGCCACTGATATTATTGGTCAAGCTGCTTTTGGTACTGACTTTGGTTTGTCTGGTAAACCAGTTGTTCCAGATGATGATATGAAGGGTGTTGATGTTGTTGTTGGTGATGCTGCTAAAGCTAAAGCTTCTTCTTCCGAATTCATCAACATGCATATCCATTCCACCACCTCATTGAAGATGGATTTGTCAGGTTCTTTGTCTACTATCGTTGGTGCTTTGGTTCCATTCTTGCAAAATCCATTGAGACAGGTTTTGTTGAGAGTTCCAGGTTCTGCTGATAGAGAAATCAATAGAGTTAACGGTGAGTTGAGAAGAATGGTTGATGGTATCGTTGCTGCTAGAGCTGCAGAAAGAGAAAGAGCACCAGCTGCTACTGCTGCTCAACAACATAAGGATTTTTTGTCCGTTGTTTTGGCTGCCAGAGAATCTGATGCTTCTACAAGAGAATTACTGTCCCCAGATTATTTGTCTGCTTTGACCTACGAACATTTGATTGCTGGTCCAGCTACTGCAGCTTTTACATTGTCATCTGTTGTTTACTTGGTTGCTAAGCACCCAGAAGTTGAAGAAAAGTTGTTAAGAGAAATGGATGCCTTTGGTCCAAGAGGTTCTGTTCCAACTGCTGATGACTTGCAAACTAAGTTTCCTTACTTGGATCAGGTCGTCAAAGAATCTATGAGGTTGTTTATGGTTTCCCCATTGGTTGCAAGAGAAACTTCTGAAAGAGTTGAAATTGGCGGTTACGTTTTGCCAAAAGGTGCTTGGGTTTGGATGGCTCCAGGTGTTTTAGCAAAAGATGCTCATAATTTTCCCGATCCAGAGTTGTTTAGACCAGAAAGATTTGATCCAGCTGGTGACGAACAAAAGAAAAGACATCCATACGCTTTCATCCCATTTGGTATTGGTCCTAGAGTATGCATTGGTCAAAAGTTCGCTATCCAAGAAATCAAGTTGGCCATTATCCACTTGTACCAACACTACGTTTTTAGGCATTCTCCCTCAATGGAATCACCATTGGAATTTCAATTCGGTATCGTCGTTAATTTCAAGCACGGTGTTAAGTTGCACGTTATCAAAAGACACGTTGAGAACAACTAA |
| *SbMAX1c* | ATGGAAATTGCCTTGACTGTTTCCGCTGTTTCTCATCAATCTGTTCCAGTTTTGGTCCTGATCTCTTTCTTGTCTTTGTTCTCTGCTTTCCTGATCTACTTCTATGCTCCATTGTGGTCTGTTAGAAGAGTTCCAGGTCCACCAACTAGATTTCCAATTGGTCACTTGCATTTGTTGGCTAAGAATGGTCCAGATGTTTTCAGAGCTATTGCCAAAGAATACGGTCCAATCTTCAGATTCCATATGGGTAGACAACCATTGGTTATCGTTGCTAATGCTGAATTGTGCAAAGAAGTCGGTATCAAGAAGTTCAAGGACATCAGAAATAGATCTACTCCACCACCATCTATCGGTTCATTGCATCAAGATGCTTTGTTCTTGACTAGAGATTCTACTTGGTCTGCTATGAGATCTACCGTTGTTCCATTATATCAACCAGCTAGATTGGCTGGTTTGATCCCAGTTATGCAATCCTACGTTGATATTTTGGTTGCTAACATTGCTGGTTGGACTGATCAAGATTGCATTCCATTTTGCCAGTTGTCTTTGAGAATGGCCATCGATATTATTGGTAAGACCGCTTTCGGTATCGAATTCGGTTTGTCTAAAAATGCTGCCGGTGGTGGTGGCGAAACTGAAGGTGGTGAAGGTGATGATAATGTCAGGGAATTCTTGAAAGAGTACAAGAGGTCTATGGAATTCGTCAAGATGGACTTGTCATCTTCCTTGTCTACTATCTTGGGTTTGTTTTTGCCATGTGTTCAAACTCCATGTAAGAGGTTGTTGAGAAGAGTACCTGGTACTGCTGATTACAAGATGAACGAAAATGAGAGAAGATTGTGCTCCAGAATCGATGCTATTATTGCTGGTAGAAGAAGAGATAGAGCTACTAGACGTAGAGGTGGTGATGGCGTTTCTGAAGATGATGCTGCTCCATTAGATTTCATTGCTGCTTTGTTGGATGCTATGGAAAATGGTGGCGGTGCTAAAGATTTTGCTTTGGCTGATAGACATGTTAGAGCTTTGGCTTACGAACATTTGATTGCAGGTACAAAGACTACCGCTTTCACTTTGTCATCTGTCGTTTACTTGGTTTCTTGCCATCCAAGAGTTGAAGAAAAGTTGTTGAGGGAAGTTGATGGTTTTGCTCCAAGACATGGTAGGGCTCCAGATGCTGATGAATTACAATCAAGATTCCCATACTTGGACCAGGTTATCAAAGAAGCTATGAGGTTCCATTTGGTGTCTCCATTGATTGCTAGACAGACTTCTGAAAGGGTTGAAATTGGTGGTTACGTTTTGCCAAAAGGTGCTTATGTTTGGTTGGCTCCAGGTGTTTTGGCTAGAGATGCTGCACAATTTCCAGATCCAGAAGAATTCAGACCAGAAAGATTTGCTCCTGAAGCTGAAGAAGAAAGAACTAGACATCCATACGCTCATATTCCTTTTGGTGTTGGTCCAAGAGCTTGTATTGGTCATAAGTTCGCTTTACAACAAGTTAAGTTGGCCGTTGTTGAGTTGTACAGAAGATACACTTTTAGACATTCCCCAGCTATGGAATCCCCATTGCAATTTGATTTCGATTTGGTTTTGGCCTTCAGACACGGTGTTAAGTTGAGAGCTATTAGAAGGTCTTAA |
| *SbMAX1d* | ATGGAAATGGCTGGTGCTGCTGGTACTGCTGAAACTTGGTTGCCATATGTTACTACTGCTGCTTCTTGTGCTGTTGCTGTTTTTTTCTTGTTGTACTTCTATGCTCCACAATGGGCTGTTAGAGGTGTTCCAGGTCCACCAGCTTTGCCAGTTGTTGGTCATTTGCCATTATTGGCTAGACATGGTCCAGATATTTTTGGTTTGTTGGCTAAAAAGTACGGCCCAATCTTTAGATTCCATTTGGGTAGACAACCATTGGTTATAGTTGCTGATCCAGAATTGTGTAGAGAAGTTGGTGTTAGACAGTTCAAGTTGATCCCAAATAGATCTTTGCCAGCTCCAATTGCTGGTTCTCCATTGCATCAAAAGGGTTTGTTTTTTACCTCCAGAGATGAGAGATGGTCTGCTATGAGAAACACCATCATTAGCTTGTACCAACCATCTCATTTGGCTGGTTTGGTTCCAACTATGCAAAGATGTATTGAAAGAGCTGCTGACGCTATTTTGGCTCCAGGTGTTCAACAAAATGGTGATGGTGATGTTGACGTCGATGTTGATTTTTCCGATCTGTCTTTGAAGTTGGCCACCGATATTATTGGTCAAGCTGCTTTTGGTGTTGATTTCGGTTTGACTGCTTCTGGTGATCCAGGTGGTGAAGCTGCTGAATTCATTAGAGAACACGTTCATTCTACCACCTCATTGAAGATGGATTTGTCTGCTCCATTGTCTGTTGCTTTGGGTTTAGTTGCTCCAGCTTTACAAGGTCCAGTTAGAAGATTATTGTCTAGAGTTCCAGGTACAGCTGATTGGAAAGTTGCTAGAACTAATGCTAGATTGAGAGCCAGAGTTGATGAAGTTGTTGCTGCTAGAGCAAGAGCTAGAGAAAGACGTAGACACGGTGAAGCTAGAACAAAGGATTTTTTGTCAGCTGTTTTGGATGCCAGAGATAGATCTGCTGCTTTGAGAGAATTATTGACCCCAGATCATGTTTCTGCTTTGACCTATGAACATTTGTTAGCTGGTTCTGCTACTACCGCTTTTACTTTATCTTCTGCCGTTTATTTGGTTGCCGGTCATCCAGAAGTTGAAGCTAAGTTGTTGGCTGAAGTTGATGGTTTTGGTCCAAGAGGTGCTGTTCCAACTGCTGATGACTTGCATCATAGATTTCCATACTTGGATCAGGTTATCATGGAAGCCATGAGATTCTATACTGTGTCTCCATTGATTGCCAGAGTTACCTCTAGAAGAACTGAATTAGGTGGTCACGAATTGCCAAAAGGTACTTGGTTGTGGATGGCACCTGGTGTTTTATCTAGAGATGCTGCTTCATTTTTTCCAGATCCTGGTGCTTTTAGACCAGAAAGATTTGATCCAGCTTCCGAAGAACAACGTGGTAGACATCCATGTGCTCATATTCCTTTTGGTATTGGTCCTAGAGCTTGTGTTGGTCAAAGATTTGCCTTGCAAGAATTGAAGTTGTCCATGGTCCACTTGTACCAGAGATTCTTGTTTAGAAGATCCCCACAAATGGAAAGTCCACCAGAATTACAATTCGGTATCGTCCTGAATTTTAAGAACGGTGTTAAGTTGGTTGCTGTTGAAAGATGTGCTGCTATGTCTTGA |
| *LGS1* | ATGAACGTCCAAGAAAGGCGTAAAGAATTGGAAGAAAGATCTTCTACTACCTTGGGTCACTTGCATACCATTAGAAATACTCCAGCTGGTTCTTCTATGTCTACTACTACTTGTTATTCTGCTCCAGCTGCTGTTGTTCCAGGTGCTGGTGGTGAAGTTGCAGTTGTTACTGCTGTTGCTTCTGAAGCTGGTGCTGCTGCTGCACATGATCAATCTAGAAAAAAGAAGAACAACCACAGGTCCTTGTACGCTAATTTGCCAGCTGCAGAAATTATCGATTCTTTGCCATTGGAAACTAGGTTCCCAGTTCCACATAGATTATATGGTGGTTTTTGGAAGGCCGAGTTCTTGTTGAAAGGTATGGCTGCAGCTGCTGCTAGAACTACTTCTTGTTTTGAATTCGAGCCAAATCCTTCCGATATTTTCTTGGCTTCATTGCCAAAATCTGGTACTACTTGGTTGAAGGCTTTGGCTTTTGCTACTTTGAACAGAAGAACTCATCCACCATCTAATGCTGATGGTCAACATCCATTTTCTCATAGAAACCCACATGACTGCGTTTCGTTCTTGGAATTGATGATGATTCAAGGTGTTGATGCTGCTGCCGCCGATGACGATGCAGGAGCTCCAAGATTAATCGCGACTCATTTGCCTTGGTCCTGGTTGCCTCCAGCCATAACGGCTGGTGAAGGTCAAGGCGGGGGTTCTTCATCTAGAGGTAGAGGTTGTAGAATCGTTTATGTCTGTAGAGAACCTAAGGACGTCTTGGTTTCTTACTGGACTTTTTCTGTTAAGGCTGCTGCAAAATTTGCTGCCGCTGCAGCCGCTGGTGGCGACGATGACGGTGGTGGTGGTAGAGAATCTGCTGCAGCTTCTTTGACTACATCTTTTGAAGAAGCTTTCGAGTTGTTCTGCGAAGGTAGATTTCCAGGTGGTCCACATTGGTTGCATGCTTTGGAATTTTGGAGAGAATCTCAAAGACGTCCAGATGAAGTTTTGTTCTTGAGGTACGAAGATATGTTGAGAGATCCAGTTGGTAACTTGAGAAAGTTGGCTGCTTTTATGGGTTGTCCATTCTCTGCTGAAGAAGAAACTGCTGGCGGAGGTGGTGGCGTTGTTGATCAAATAGTTGAATTGTGCTCCTTGGAGAACTTGAAGTCTATGGATGTTAACAAGAACGGTACTACCACTGTTTTGGGTGTTACTAATGATGCCTTTTTCAGAAAAGGTAAGGTTGGTGACTGGAAGAATTACATGACTCCAGATATGGCTGCTAGATTGGATAAGGTTGTCGAAGAAGCTACTAGAGGTTCTGGTTTGACTTTCGCTGATTCCATTTCTGTCTGA |
| *LGS1-2* | ATGAACGTCCAAGAAAGGCGTAAAGAATTGGAAGAAAGATCTTCTACTACCTTGGGTCACTTGCATACCATTAGAAATACTCCAGCTGGTTCTTCTATGTCTACTACTACTTGTTATTCTGCTCCAGCTGCTGTTGTTCCAGGTGCTGGTGGTGAAGTTGCAGTTGTTACTGCTGTTGCTTCTGAAGCTGGTGCTGCTGCTGCACATGATCAATCTAGAAAAAAGAAGAACAACCACAGGTCCTTGTACGCTAATTTGCCAGCTGCAGAAATTATCGATTCTTTGCCATTGGAAACTAGGTTCCCAGTTCCACATAGATTATATGGTGGTTTTTGGAAGGCCGAGTTCTTGTTGAAAGGTATGGCTGCAGCTGCTGCTAGAACTACTTCTTGTTTTGAATTCGAGCCAAATCCTTCCGATATTTTCTTGGCTTCATTGCCAAAATCTGGTACTACTTGGTTGAAGGCTTTGGCTTTTGCTACTTTGAACAGAAGAACTCATCCACCATCTAATGCTGATGGTCAACATCCATTTTCTCATAGAAACCCACATGACTGCGTTTCGTTCTTGGAATTGATGATGATTCAAGGTGTTGATGCTGCTGCCGCCGATGACGATGATGCAGATGATGCAGGAGCTCCAAGATTAATCGCGACTCATTTGCCTTGGTCCTGGTTGCCTCCAGCCATAACGGCTGGTGAAGGTCAAGGCGGGGGTTCTTCATCTAGAGGTAGAGGTTGTAGAATCGTTTATGTCTGTAGAGAACCTAAGGACGTCTTGGTTTCTTACTGGACTTTTTCTGTTAAGGCTGCTGCAAAATTTGCTGCCGCTGCAGCCGCTGGTGGCGACGATGACGGTGGTGGTGGTAGAGAATCTGCTGCAGCTTCTTTGACTACATCTTTTGAAGAAGCTTTCGAGTTGTTCTGCGAAGGTAGATTTCCAGGTGGTCCACATTGGTTGCATGCTTTGGAATTTTGGAGAGAATCTCAAAGACGTCCAGATGAAGTTTTGTTCTTGAGGTACGAAGATATGTTGAGAGATCCAGTTGGTAACTTGAGAAAGTTGGCTGCTTTTATGGGTTGTCCATTCTCTGCTGAAGAAGAAACTGCTGGCGGAGGTGGTGGCGTTGTTGATCAAATAGTTGAATTGTGCTCCTTGGAGAACTTGAAGTCTATGGATGTTAACAAGAACGGTACTACCACTGTTTTGGGTGTTACTAATGATGCCTTTTTCAGAAAAGGTAAGGTTGGTGACTGGAAGAATTACATGACTCCAGATATGGCTGCTAGATTGGATAAGGTTGTCGAAGAAGCTACTAGAGGTTCTGGTTTGACTTTCGCTGATTCCATTTCTGTCTGA |
| *TaSOT* | ATGAACGCTGCTTTGACTGTTGCTGTTGCTGGTGAAGAAATTGATGAAGCAGTAGCAGGCGAAGAAATAGACGAAGCTGCTTCTAGAGCACAAGCTGATATGTCTGAAATCATGTCATCTTTGCCAAGATGCCCAGTTTACTTGACTCATCATTATAGAGGTTTCTGGATCAGGGAATTCGTCTTGAAAGGTATGGCTGCTGCTCAAGCTTCTTTTGAACCTAGACCAACTGATGTTTTCTTGGCTTCTTGTCCAAAATCTGGTACTACTTGGTTGAAGGCTTTGGCTTTTGCTACTTTGAATAGAGCTACTCACTTGCCATCTGATTCTAACCATCCATTGTGTCATAGAAACCCACATGATTGTGTTGCTTTCTTGGAAACTAGACCAGTTCCAGAAACTATGGCTTTGCCATCTCCAAGATTATTGGCTACTCATATCCCATGTTCTTTGTTGCCATCTAGAATTACCGAATGCGGTAGAGTTGTTTATGTTTGTCCAGAACCTAAGGATGCCTTGGTTTCTTTTTGGATCTACAACAACAAGATCGCCCCAATGTTGAGAAGAAAGTTTGGTTTGGAATCTCCATCACCAACTTTCGAAGAAGCTTTTGAGTTGTTCTGTGAGGGTCAATCTTCATTTGGTCCACCTTGGAGACATGCTTTGGAATATTGGGAAGAATCTAGAAGAAGGCCAGGTAAGGTTTTGTTCTTGAGATACGAAGATATGTTGCAAGATCCAACTGGTAACACTAAGAATTTGGCTGCTTTTATGGGTTGTCCATTCTCTTGTGCTGAAGAAGAAGCTGGCGTTGTTCAAGAAATAGTTCAATTGTGCTCCTTCGAGAAGTTGAAGTCATCTGAAGTTAACAAGAACGGTTCCTCTGCTATGATGGGTGTTAACAATGATGTCTACTTTAGAAAGGGTGCTGTTGGTGATTGGAAGAATTATATGACTCCAGAAATGGCTGCCAGGTTGGATAAGATAGTTGAAGAAGCCTTACAAGGTTCTGGTTTGACTTTCGGTATTTCCATGTGA |
| *ZmSOT* | ATGTACCATCAAACTACCTCCAGACCACAACAAAGACCATCTACTAGACAACCACATCAAATTCATGCTGTTTTGTTGCCAGATTTGCCAGTTTCTCCAATTGCTTCTACAAGACGTAGAGCTAGAGGTATTCATTTGTTGCACTTGCAATTGTGCTACTGTAGATCTTGTTGTAACAGAGGTGAAAAGAGAGATCAATTCGCTGGTACTAATACCGCTTGCTGTCATATTTCTGGTATTTTCTTTAGACAAGGCTGGGGTTTGAAAACCGTTGCTGTTTCTTGGGGTTGGGGTTTGTTTCAAGCTGCTGCTTCTAATTGGACTATGATGGCTTCTAGACAGTCTGAAAACAACGCTCAAGAAGAAACTTCTCCATTGACTACTCCAAACGCTAACATTGCCAGAATTATTCCATCTTTGCCATTGGAAACTAGGTGGCCTCCATTTCCATTGAGAAGATATGCTAATTTCTGGTTGCCAGAGGTCACTTTGAAAGAAGGTGTTCCAGGCGTTCATTCTTGTTTTGAACCTAGACCAACTGATGTTTTCTTGGCTTCTTTTCCCAAATCTGGTACTACTTGGTTGAAGGCTTTGGCTTTTGCTACTTTGAAGAGATCTACTCATCCACCATTCGATGATGATCACCCATTGAGACATTGCAATCCACATGATTGTGTCAGGTTTTTGGAGTTGGGTTTCAATCAACAAAAGGACGAATTGGAAGCTTTGCCATCTCCAAGAGTTTTGGCTACTCATTTGCCATACTCATTATTGCCAGGTTCTATTACTGGTGATGGTGAACATTCTGGTTGTAGAATCGTTTATGTCTGCAGAGAACCTAAGGATACCTTGGTTTCTTACTGGTTGTTTACTAGAAAAGCTGCTCCAGCTTGTGGTGTTGATGCTAGATCTTTTACTATTCAAGAAGCCTTGGAGTTGTTCTGTGATGGTAGATGTCCAGGTGGTCCACAATGGAATCATGTTTTACAATACTGGAAAGAGTCCGTTAGAAGGCCAGATAGAGTTTTGTTTTTGAGGTACGAAGAAGTCTTGATCGAACCTGAAGCTCATGTTAGAAAGTTGGCTAATTTCATGGGTTGTGGTTTCTCTGAAGAGGAAGAAGAAAGAGGCGTTGTTTCTACTATCGTTGAATTGTGCTCTTTGGGCAAGTTGAGAGATATGGAAGTTAACAGAAACGGTTCTACCAGATTGGGTACTAAGAACGAATCATTCTTCAGAAAAGGTGTTGCTGGTGATTGGTCTAATCATATGACTCCAGAAATGGCTCACTCCTTGGATAAGGTTGTTGAAGATGCTTTACAAGAGACTGGTTTCACTTTCTCTTCTACCACTTGA |
| *MlSOT* | ATGAGTACAACCACCTGTTATGATTCCGCGACAGCGGTACCTGCAGGAGGAGAGGTGGTTACCGCGGTACCAAGTGAGGAGGCGGCGGCTGCTGCTGTACACCAGAGTAGGAAAAACTTATCCTTGTACGCCAATCTGCCGGCTGCGGAGATCATCGACTCATTACCGTTGGAAACGCGTTTCCCTGTACCTCACAGGCAATATGGAGGGTTCTGGAAGGCCGAGTTTCTGTTGAAGGGGATGGCGGCTGCAGCAACCAGGTCCACTTGTTTCGAACCAAACCCCTCTGACATATTCTTGAGCAGCCTTCCGAAGTCCGGCACGACCTGGCTTAAGGCCTTGGCATTCGCTACACTGAATCGTGGCACGCATCCACCCTCTAATGCTGACGGGCAACATCCCTTGAGCCATAGAAACCCGCACGACTGCGTTTCATTCCTGGAGTTAATGATGATTCAGGGCGTTGATGCGGCCGCGGCTGCAGGAGCGTCTGGAGAAGAGCGTGGTTCCCCCCCACCGAGACTAATTGCGACGCACTTACCCTGTTCTTGGCTTCCCCCGGCCATAGTGACGGGCTCAGGATGCAGGATCGTATACGTGTGTCGTGAGCCGAAGGACGTCCTAGTTTCTTATTGGACGTTTTCCGTTAAAGCGGTCGCGAAGTTTGCAGCAGCAGCGGCCGCAGGAGGTGATGGTGACGGGGATGGGGGAGGTGGACGTGAAGCTGCCGCCGCAAGCGCGGGGGGGCTTACTTCATTTGAAGAAGCTTTCGAACTATTCTGTGAGGGAAGATTCCCTGGTGGGCCACACTGGTTGCATGCCCTTGAATATTGGCGTGAGTCTCAGAGGAGGCCGGACGAGGTGCTATTTTTGAGATACGAGGACATGCTGAGGGACCCAGTAGGGAACTTGAAAAAACTGGCCGCATTCATGGGGTGTCCGTTCTCTGCAGAGGAAGAGAAAGCAGGCGGGGTAGTAGATCAAATCGTAGAGTTGTGTAGCCTGGATAATTTAAGGAGTATGGAAGTCAATAAGAATGGCAGCACAACCGTATTAGGTGTGACGAATGATGCCTTCTTCCGTAAGGGGCAGGTCGGCGACTGGCGTAATTACATGACTCCCGACATGGCTGCTAGATTAGACAAAGCGGTGGAGGAAGCGACAAGAGGGAGCGGCCTGACGTTCGCAGACAGTATTGAAGTATAG |
| *ZmMAX1a* | ATGGAAATGGCTGGTGCTGCTGGTACTGAAGCTTGGTTGCCATATGTTACTACTGTTGCTTCTTGTGCTGTTGGCGTTTTTTTCTTGTTGTACTTTTATGCCCCACATTGGAGAGTTAGAGATGTTCCAGGTCCACCAGCTTTGCCAGTTGTTGGTCATTTGCCATTATTGGCTAGACATGGTCCAGATGTTTTTGGTTTGTTGGCTAAAAAGTACGGTCCAATCTTCAGATTCCATTTGGGTAGACAACCATTGGTTATAGTTGCTGATCCAGAATTGTGTAGAGAAGTTGGTGTTAGACAGTTCAAGTTGATCCCAAATAGATCTTTGCCAGCTCCAATTGCTGGTTCTCCATTGCATCAAAAGGGTTTGTTTTTCACCAGAGATGAGAGATGGTCTGCTATGAGAAACACCATCATTAGCTTGTACCAACCATCTCATTTGGCTGGTTTGGTTCCAACTATGCAACATTGCATTGAAAGAGCTGCTGACGCTATTCCAGCTATGGTTGTTCAAGAAAATGGTCAGGTTGATTTCTCCGACTTGTCTTTGAAATTGGCCACCGATATTATTGGTCAAGCTGCTTTTGGTGTTGATTTCGGTTTGACTGCTTCTGGTCCAGGTTGTGAAGCTGCTGAATTCATTAGAGAACACGTTCATTCTACCACCTCATTGAAGATGGATTTGTCTGCTCCATTGTCCGTTGTTTTGGGTTTAGTTGCTCCAGCTTTACAAGGTCCAGTTAGACATTTGTTGTCTAGAGTTCCAGGTACTGCTGATTGGAGGGTTGCTAGAACTAATGCTAGATTGAGAGCTAGAGTTGACGAAATCGTTGTTTCTAGAGCAAGAGGTAGAGGTCAACATGGTGAAAGAAGAAAGGATTTCTTGTCTGCTGTTTTGGATGCCAGAGATAGATCTGCTGCTTTGAGAGAATTATTGACCCCAGATCATGTTTCTGCTTTGACCTACGAACATTTGTTAGCTGGTTCTGCTACTACCGCTTTTACTTTATCTTCAGCCGTTTATTTGGTTGCCGGTCATCCAGAAGTTGAAGCTAAGTTGTTGGCTGAAGTTGATGCATTTGGTCCACATGGTGCTGTTCCAACTGCTGATGACTTGCAACATAGATTCCCTTATTTGGATCAAGCTTCTGATACCACCATGCAAAGACACGTTATCAAAGAAGCTATGAGGTTCTACACTGTGTCTCCATTGATTGCTAGAGTCACTTCTAGACAAACTGAATTAGGTGGTCATACCTTGCCAAAAGGTACTTGGTTGTGGATGGCTCCAGGTGTTTTATCTAGAGATGCTGCTAATTTTGAAGATCCAGGTGCTTTCAGACCAGAAAGATTTGATCCAGTTTCCGAAGAACAAAGACGTAGACATCCATGTGCTCATATTCCTTTTGGTATTGGTCCAAGAGCTTGTGTTGGTCAAAGATTTGCCTTGCAAGAGGTTAAGTTGAGTATGTTGCACTTGTACAGAAGGTTCTTGTTTAGAAGATCCCCAAGAATGGAATCACCACCAGAATTACAATTCGGTATCGTCCTGAATTTTAAGAAGGGTGTTAAGTTGGTTGCTGTTGAAAGATGTGCTGCTATGCCATTGTGA |
| *ZmMAX1b* | ATGTTGGCTTCTGCTGTTTTGAGAGCTATGGAAGAATGTACTTTTACCTCTGCTGCTATGGCTGTTGGTTTTTTGTTGGTTGTTTACTTGTACGAGCCATACTGGAAGGTTAGACATGTTCCAGGTCCAGTTCCATTGCCATTTGTTGGTCACTTGCATTTGTTAGCTAGACATGGTCCTGATGTTTTCTTGGTTTTGGCTAAAAAGTACGGTCCAATCTTCAGATTCCATATGGGTAGACAACCATTGGTTATCGTTGCTAATGCTGAATTGTGCAAAGAAGTCGGCATCAAAAAGTTCAAGTCTATGCCAAATAGGTCCTTGCCATCTGCTATTGCTAATTCCCCAATTCATTTGAAGGGTTTGTTCTCCACTAGAGACTCTAGATGGTCTGCTTTGAGAAACATCATCGTGTCTATCTACCAACCATCTCATTTGGCTGGTTTGATTCCATCTATGCAATCCCATATTGAAAGAGCTGCTACCAATTTGGATGATGGTGGTGAAGCTGAAGTTGCTTTTTCTAAATTGGCTTTGTCTTTGGCCACCGATGTTATTGGTCAAGCTGCTTTTGGTGCTGATTTTGGTTTGACTACAAAACCAGCTGCTCCACCACCACATCATGGTCCACCAAGACAACATGGTGAAGAGGATGGTGATGGTTCTCATTCTACTAGATCTTCCGAATTCATCAAGATGCATATCCATTCTACCACCTCATTGAAGATGGATTTGTCTGGTTCTTTGTCTACCATCGTTGGTACTTTGTTGCCAGTTTTACAATGGCCTTTGAGACAGTTGTTGTTGAGAGTTCCAGGTGCTGCTGATAGAGAAATTCAACGTGTTAATGGTGCTTTGTGCAGAATGATGGATGGTATTGTCGCAGATAGAGTTGCTGCAAGAGAAAGAGCACCACAAGCTCAAAGACAGAAGGATTTTTTGTCAGTTGTTTTGGCTGCCAGAGATTCTGATGCTGCTGCTAGAAAGTTGTTGACTCCAGATTATTTGTCCGCTTTGACCTACGAACACTTGTTAGCTGGTTCTGCTACTACTGCTTTTACTTTGTCATCTGTCTTGTACTTGGTTGCCCAACATCCAAGAGTTGAAGAAAAGTTGTTAAGAGAAGTTGATGCTTTCGGTCCACCTGATAGAGTTCCAACTGCTGAAGATCTACAATCCAGATTTCCATACACCGACCAAGTCTTGAAAGAATCTATGAGGTTCTTCATGGTTTCCCCATTGGTTGCTAGAGAAACTTCTGAACAAGTTGATATTGCCGGTTACGTTTTGCCAAAATCTACTTGGGTTTGGATGGCTCCAGGTGTTTTAGCAAAAGATCCAGTTAATTTTCCAGAGCCAGAGTTGTTTAGACCAGAAAGATTTGATCCAGCTGGTGATGAACAAAAAAGAAGGCATCCATACGCTTTCATTCCATTTGGTATTGGTCCAAGAATCTGCATCGGTCAAAGATTCTCTATCCAAGAAATCAAGTTGGCCTTGATCCACTTGTACAGACAATACGTTTTTAGGCACTCTCCCTCTATGGAATCACCATTGGAATTTCAATTCGGTGTCGTCTTGAACTTCAAACACGGTGTTAAGTTGCAGTCCATCAAGAGACATAAGTGCTGA |
| *ZmMAX1c* | ATGGAAATCACCGCTTCCTGTGATGACGGTGCCGTCACTGCCGGTGCTGTCTCTGGTTTATTGTTGGCTTCTGTCTTGTCTTTGTTCGGTGCTTTCTTGGTTTACTTCTACGCCCCATTCTGGTCCGTTCGACGTGTTCCAGGTCCTCCTGCCAGATTCCCAATCGGTCATTTGCATTTGCTCGCCAGAAACGGTCCAGATGTCTTCAGAGCTATTGCCAAGGAATACGGTCCAATCTTCAGATTCCACATGGGTAGACAACCATTGGTCATTGTTGCTAATGCTGAATTGTGTAAGGAAGTCGGTATCAAGAAATTCAAGGATATTCCAAACAGATCAACTCCTCCACCATCTATTGGTTCTTTGCACCAAGATGCTTTATTCTTGACTAGAGACTCCACCTGGTCTGCTATGAGATCAACTGTCGTTCCATTGTACCAACCAGCTAGATTGGCTGGTTTGATTCCAGTTATGCAATCTTACGTTGACACTCTTGCTGCTAACATTGCTGCTTGTCCAGATCAAGACTGTGTTCCATTCTGCCAATTGTCTTTGAGAATGGCTATTGACATCATCGGTAGAACTGCTTTTGGTATTGAATTTGGTTTATCCAAGAACGCTGCCGGTACTGGTTCCTCTTCTTCTGAATCTCCAGGTGGTGGTGAAGGTGAAGGTGACGTCAGAGAATTCTTAAGAGAGTATAAGAGATCCATGGAATTCGTTAAGATGGATTTGACCTCTTCCTTGTCTACCATCTTGGGTTTGTTTTTGCCATGCGTTCAAACTCCATGTAAGAGACTTTTGAGAAGAGTCCCAGGTACTGCTGACTACAAGATGGACCAAAACGAAAGAAGATTGTGTTCTAGAATTGATGCTATCATTGCTGGTCGCAGACGTGACAGGGCTACCAGAAGAAGATGTGGTCCGGGTGCAGCTCCAGCTCCAGCTCCTTTGGATTTCATCGCTGCTCTTCTGGATGCTATGGAAAGCGGGGGAGGTGGTGGCGGTGGTGCTGGTGCCAACAAGGACTTCGCTCTAGCAGACAGACACGTTAGAGCTTTGGCCTACGAACACTTAATTGCTGGTACCAAGACTACCGCTTTCACCTTGAGTTCTGTTGTGTACTTGGTTTCTTGTCACCCATTGGTAGAAGCTAAGTTGTTGAGGGAATTAGACGGTTTCGCGCCAAGAAGAGGTAGAGGTAGAGCTCCAGATGCTGATGAATTGCAATCCGGTTTTCCATACCTAGACCAAGTTATCAAGGAAGCCATGAGATTCTATGTTGTTTCCCCATTGATCGCTCGTCAAACCTCCGAAAGAGTTGAAATCGGTGGTTACGTTTTGCCAAAGCAAGGTGCTTACGTCTGGTTGGCCCCAGGTGTTTTAGCAAGAGATGCCGCTCAATTCCCAGACCCAGAAGAATTCAGACCAGAAAGATTTGCGCCAGAAGCTGAAGAAGAAAGAGCTCGTCACCCATACGCTCACATCCCATTCGGTGTTGGTCCAAGAGCCTGTATCGGTCACAAGTTCGCCTTGCAACAAGTCAAATTGGCCGTCGTCGAATTGTACAGAAGATACGTCTTTCGTCATTCTCCATCCATGGAATCCCCAATTCAATTCGACTTCGACTTAGTCTTAGCTTTCAGACACGGTGTCAAATTGCGTGCTATCAGAAGAGGTTGA |
| *SbCYP722B* | ATGGATGACATGCACTCTCAATTGCAAGCTGCTGGTGCTGCTTGTCAACAATCTAATTCTTTGTTGTTGCCACCACCAGCTGCTGATAGACCTTGTTCTTCTTCATCTTCATCCTCGTTGTCTTTGTTGGGTACAGCTGCTGCTGCATGTTTGTTTTTGTCTGCTGCTATCTACTGCATCGTCGTTATTATCGTTACTACCTCCTCTAAGCAGAACATCAACAACAGATTGATCAGGCGTTTGTTGAAGTTCAAGGGTAGAAGATCTAAGAACGACAGAAGAAGAGACTACAACAACAATGCTGCTCCACCACCACCACCTCCAGGTAGAGGTTCTTCTTGGTGGTGGTCAGTTGTTGAAACTTTGGCTTTTGTTTCCGCTAACAGATCTGGTAGAGGCTTGTATCATTTCGTTGAAGCTAGACATAGAAGATACGGTCCACCATGTTTTAGAACTGCTTTGTTAGGTGCTACCCACGTTTTTGTTTCTTCACCAGATGCTGCTAGAAGTTTGTTGGCTGATGCTGGTGGTTTTTCTAAGAGATACGTTAGAACCGTTGCCGAATTATTGGGTGAACATTCTTTATTGTGCGCTTCCCATGATGCTCATAGAGCTTTGAGAAGGGCTGTTGCTCCTTTGTTTAATGCTCAAGCTACTGCTTCTTTGGCTGCTAATTTTGATGCTTTGGCCAGAAGAATTATCACCAGAGATTGGGCTGCTAAAACTACTGCTGTTGTTGTTTTGGATGCTGCTTTGGATGTTACCTTCGAAGCTATTTGCGATATGTTGATTGGTAGAACTACCACCTTGAAGCGTAGAAGATTACAATCTGATGTTTTGGCTGTTACCAGAGCTATGTTGGCTTTTCCATTGAGATTGCCAGGTACTAGATTTCATGCTGGTTTGAGAGCCAGAAAAAGAATCATGGATGTCTTGAGACAAGAAATCGCTTCCAGACAAAGAAACATCATGGATATGGAAGAAATGGAAGAGGATGATTCCAAGCACGACAATGATTTCTTGCAGTCCTTGTTGTTATTGAGGCGTAGAAAGATGAAGTCCTCTCAACAGCAACAATCTCCATCTAACTCTAACGATCATTTGTTCTTGACCGACGATCAAATCTTGGATAACATCCTGACCTTGATTATTGCTGGTCAAGTTACTACAGCTTCTGCTATTACTTGGATGGTTAAGTACTTGGCCGATAACAAGGATTTCCAAGAAACCTTGAGATCCGTGCAATTGGAAATGGCTTTGAAACACCAACATGGTGATTCTGATGGTCCTTTGACTCTGCAACATTTGAACTCTATGGAATTGGCTTACATGACCGTCAAAGAAAGTTTGAGAATGGCCTCTATCGTTTCCTGGTTTCCAAGAGTTGCTTTGGAAGATTGTCAAGTTGCTGGTTTTCATATCAACAAAGGTTGGATCGTTAACATTGATGCTAGAGCCTTGCATTATGATGCTACCTTGTATGATAACCCAACCATGTTTGATCCATCCAGATTCAAAATGGGTGATGGTAGAAGGTGA |
| *SbCYP728B1* | ATGGCTGCTTCTGTTGGTGTTGCTTTGTTGGTTGCTTTTTTGACTCCAGTTGTTGTCTACTTGTTGACCAGACATCCAAACAAAAAACCATTGCCAGGTAATTTGCCACCAGGTTCTTTGGGTTTGCCAATGATTGGTCAATCTTTGGGTCTATTGAGAGCCATGAGATCTAATACTGGTGAAAGATGGTTGAGAGATAGAGTTGATAGATACGGTCCAGTCTCTAAGTTGTCTTTGTTTGGTGTTCCAACTGTTTTCGTTACTGGTCCAGCTGCTAACAAATTGGTTTTTGCTTCTGATGCTTTGGCTCCAAAACAACCTAGATGTTTGCCTTTGATTTTGGGCAGAAGAAACATCTTGGAATTGGTTGGTGATGATTACAGACGTGTTAGAGGTGCTATGATGCAATTTTTGAAGCCAGACATGTTGAGAAGATACGTTGGTACTATTGATGCTGAAGTTGCCAGACACTTGGAAGGTAGATGGGCTGGTAGAAGAACTGTTGCTGTTTTGCCATTGATGAAGTTGTTGACCTTCGATATTATTGCCACCTTGTTGTTCGGTTTGGAAAGAGGTGCTGTTAGAGAAAGATTGGCTGCTGCTTTTGCTGATATGTTAGAAGGTATGTGGTCTGTTCCATTGGATTTGCCATTCACTACTTTCAGAAAGTCCTTGAGAGCTTCTGCTAGAGCTAGAAGAGTTTTGGAAGCTACTTTGGCTGAAAAGAGAGCTAGATTGGAAAGGGGTGAAGCTTCTCCAGCTGATGATTTGGTTTCTTGTTTAGCTTCTTTGAGAGCTGAAGCTGAAGGTGATGGTGGTGAAAGGTTGTTGACTGATGAAGAAATCGTTGATAACGCCATGGTTGTTTTAGTTGCTGGTCATGATACTTCGTCTGTTTTGATGACCTTCATGATTAGACATTTGGCTGGTGATCCAGCTACATTAGCTGCTATGGTTCAAGAACATGACGAAATTGCTAAGAACAAGGCTGATGGTGAAGCCTTGACTTGGGAAGATCTACATGGTATGAGATTCACTTGGAGAGTTGCTTTGGAAACCTTGAGAATGATTCCACCAATCTTCGGTTCTTTTCGTAGAGCCATGGAAGATATTGAATTCGATGGTTACTGCATCCCAAAAGGTTGGCAAGTTTTTTGGGCTTCTTCTGTTACTCATATGGACCCATCTATTTTTCCAGATCCAGATAAGTTCCAAGCCTCTAGATTTGAATCTCAAGCTCCACCATATTCCTTTGTTGCTTTTGGTGCTGGTCAAAGATTGTGTGCTGGTATTGAATTTGCTAGAGTTGAAACCTTGGTTACCATGCATAGACTATTGAGAAGGTTTAGATGGCGTTTGTGTTGCGAAGATAAGGATAACACTTTCGTCAGAGATCCAATGCCATCTCCATTGAATGGTTTGCCTATTGAATTGCAGTCTAGAGATATGGCTTCTCCAACTCCATCTAAATCTGCTTGTGGTTTGTGA |
| *SbCYP728B35* | *ATGCACATCCCATTGGTTGAAGAATTGAGATTGGCTTCTCCAATGGACTCCTCTTTGATTTTGGCATTGATTTTAGCTGTTGCCTTGGCCTTGTTGTTGCATTTGTTGACATCTGCTAACAACAAACCTAGAAGGGCTAAACAAGTTCCACCAGGTTCTTTGGGTTTGCCAGTTATTGGTCAATCTTTGTCTTTGTTGAGAGCTATGAGAGCCAATTCTGGTGAAAGATGGATTCAAGATAGAATCCATAGATACGGTCCAGTCTCTAAGTTGTCTTTATTTGGTGCTCCAACTGTTTTGTTGGCTGGTCCAGCTGCAAACAAGTTTACATTTTTTTCAAGAGCCTTGGCCATGCAACAGCCAAGATCTGTTCAAAGAATTTTGGGTGAGAAGTCCATCTTGGAATTGGTTGGTGCTGATCATAAGAGAATTAGAGGTGCTTTGGCTGAATTCTTGAAGCCAGATATGTTGAGGTTGTACGTTGGTAAGATTGATGGTGAAGTTAGAAGGCATTTGGACGAAAGATGGGCTGGTAGAACTACTGTTACTGTTATGCCATTGATGAAGAGATTGACCTTCGACATCATCTCGTTGTTGTTGTTCGGTTTACAAAGAGGTGCTCCATTACAAGATGCTTTGGCAGCTGATTTTGCTAGAGTTATGGATGGTATTTGGGCTGTTCCAGTTAATTTGCCATTCACTGCTTTCTCCAGATCTTTGAGAGCTTCTGCTAGAGCTAGAAGATTGATTGCTGGTATTTTGAGAGAAACCAGAGCTAAGTTGGAAACTGGTGAAGCTTCTAGATCCTCTGATTTGATTGCTTGCTTGTTGTCTTTGACCGATCATCATTCTGGTGCACCTTTGTTGTCTGACAAAGAAATCGTTGATAACTCCGTTGTTGCTTTGGTTGCTGGTCATGATACTTCGTCTATTTTGATGACCTTCATGGTTAGACAATTGGCCAACGATCCAGATACTTTGGCTGCTATGGTTCAAGAACATGATGATATCGCTAAGTCCAAAGGTGATGGTGAGGCTTTGGATTGGGAAGATTTGGCTAAAATGAAGTACACTTGGAGAGTCGCTTTGGAAACCTTGAGATTAGTTCCACCTATGTTTGGTGATTTTAGAAGGGCATTGCAAGACGTTGAATTCGATGGTTACTTGATTCCAAAAGGTTGGCAAGTTTTTTGGGTTGCTTCTGTTACTCATATGGATCCAGGTATTTTTCCAGAACCAGCTAGATTTGAACCATCCAGATTCGAAAATCAATCCCCACCATGTTCATTCGTTGCTTTTGGTGGTGGTCCAAGAATTTGTGTTGGTATGGAATTCGCTAGAATCGAAACTTTGGTTACCATGCACTATTTGGTGAGAAGATTCAGATGGAAGTTGTGCTGTAAGAAGGATACTTACGCTAGAGATCCAATGCCATTGCCATTGCATGGTTTGCCAATTCAATTGGAACATAAGGTTTCTCCATGCGTCATGTGA* |
| *ZmCYP728B35* | *ATGGACTCCTCTTTGGTTTTGGCTTTGATTGCTGTTGCTTTGCCAGTTTTGTTGCACTTGTTGAAAAGAGGTAAAACTCCTTGGAGGCCAGCTGCTGCTAAATTGCCACCAGGTTCTTTGGGTTTACCAGTTATTGGTCAATCCATCGGTTTGTTGAGAGCTATGAGAGCAAATACTGCTGAAAGATGGATCTTGGATAGAATCCATAGATACGGTCCAGTCTCTAAGTTGTCTTTGTTTGGTAGACCAACTGTTTTGGTTGCTGGTTCAGCTGCTAATAGGTTCATTTTTTTCTCATCCGCTTTGGCTATGCAACAGCCAAGATCTGTTCAAAGAATTTTGGGTGACAAGTCCATCTTGGAATTGACTGGTGCTGATCATAAGAGAATTAGAGGTGCTTTGGTCGAATTCTTGAAGCCAGATATGTTGAGGTTGTACGTTGGTAAGATTGATGGTGAAGTTAGAAGGCATTTGGATGAATGTTGGGCTGGTAGATGTACTGTTACTGTTATGCCACATATGAAGAGATTGACCTTCGACATCATCAGCTTGTTGTTGTTTGGTTTGGAAAGATCCCCATTGCAAGATGCTTTGGCTGGTGATTTTGCTAGAGTTATGGATGGTATTTGGGCTGTTCCAGTTAATTTGCCATTCACTGCTTTCTCCAGATCTTTGAGAGCTTCTGCTAGAGCTAGAAGATTGATTGCAGGTATTGCTAGAGAAACCAGAGCTAAATTGGAAAGAGGTGAAGCTTCTAGATCCTCTGATTTGATAGCATGCTTGTTGTCCTTGACTGATCATTCTGGTGCTAGATTGCTATCCGAAGAAGAAATCGTTGATAACTCCATGGTTGCTTTAGTTGCTGGTCATGATACCTCTTCTATTCTGATGACTTTCATGGTTAGACACTTGGCTAATGATCCAGATACTTTAGCTGCTATGGTTCAAGAACATGACGAAATCGCTAAGAACAAAGGTGATGGTCAAACTTTGGATTGGGAAGATTTGGCTAAGATGAAGTACACTTGGAGAGTTGCTTTGGAAACCTTGAGATTGGTTCCACCAATTTTTGGTAACTTTAGAAGGGCCATGCAAGATATTGAATTCGACGGTTACTTGATCCCAAAAGGTTGGCAAGTTTTTTGGGCTGCTTCTGTTACTCATATGGATACTGGTATTTTCCATGAACCAGCTAAGTTCGATCCATCCAGATTCGAAAATCAATCTGCTGCTTCAGCTCCACCATGTTCATTTGTTGCTTTTGGTGGTGGTCCAAGAATTTGTGTTGGTATGGAATTCGCTAGAATCGAAACTTTGGTTACCATGCACTATTTGGTGAGAAGATTCAGATGGAAGTTGTGCTGTAAGAACGATACTTTCGCTAGAGATCCAATGCCATCTCCATTGCATGGTTTGCCAATTGAATTGGAACAAAAGGCTTCTCCCTGA* |

**Supplementary Table 5.** Primers used for site-directed mutagenesis of LGS1

| Primer name | Sequence (5’-3’) |
| --- | --- |
| H216A-F | TCGCGACTGCTTTGCCTTGGTCCTGGTTGCC |
| H216A-R | AAGGCAAAGCAGTCGCGATTAATCTTGGAG |
| H317A-F | CATTGGTTGGCTGCTTTGGAATTTTGGAGAG |
| H317A-R | CAAAGCAGCCAACCAATGTGGACCACCTGG |
| Y247F-F | GAATCGTTTTTGTCTGTAGAGAACCTAAGGA |
| Y247F-R | TACAGACAAAAACGATTCTACAACCTCTAC |
| K148A-F | CATTGCCAGCTTCTGGTACTACTTGGTTGAA |
| K148A-R | TACCAGAAGCTGGCAATGAAGCCAAGAAAA |

**Supplementary Table 6.** Accession numbers of MAX1 analogs used for the phylogenetic tree analysis in Figure 2 and Supplementary Figure 1. The amino acid sequences are downloadable from NCBI, except for SfMAX1, which is downloaded from https://phytozome.jgi.doe.gov/pz/portal.html.

| **Gene** | **Species** | **Size (a.a.)** | **Accession numbers** |
| --- | --- | --- | --- |
| *AtMAX1* | *Arabidopsis thaliana* | 522 | NP_565617 |
| *PtMAX1a* | *Populus trichocarpa* | 529 | XP_006372016 |
| *PtMAX1b* | *Populus trichocarpa* | 529 | XP_006382011 |
| *PhMAX1* | *Petunia x hybrida* | 533 | AEB97383 |
| *SmMAX1a* | *Selaginella moellendorffii* | 512 | AGI65366 |
| *SmMAX1b* | *Selaginella moellendorffii* | 512 | BBA85738 |
| *OsCYP711A2*  (*Os01g0700900*) | *Oryza sativa* (*rice*) | 539 | XP_015633367 |
| *OsCYP711A3*  (*Os01g0701400*) | *Oryza sativa* (*rice*) | 541 | XP_015644699 |
| *OsCYP711A4*  (*Os01g0701500*) | *Oryza sativa* (*rice*) | 516 | XP_015642272 |
| *OsCYP711A5*  (*Os02g0221900*) | *Oryza sativa* (*rice*) | 548 | XP_015626073 |
| *OsCYP711A6*  (*Os06g0565100*) | *Oryza sativa* (*rice*) | 540 | XP_015644019 |
| *SlMAX1* | *Solanum lycopersicum* (*tomato*) | 519 | XP_004245085 |
| *StMAX1* | *Solanum tuberosum* | 519 | XP_006351579 |
| *ZmMAX1a* | *Zea mays* | 537 | PWZ07057 |
| *ZmMAX1b* | *Zea mays* | 543 | ONM29770 |
| *ZmMAX1c* | *Zea mays* | 560 | XP_020407074 |
| *AmtMAX1* | *Amborella trichopoda* | 555 | XP_011626843 |
| *AcMAX1* | *Aquilegia coerulea* (*columbine*) | 539 | PIA42995 |
| *AlMAX1* | *Arabidopsis lyrata* | 522 | XP_020884785 |
| *BrMAX1* | *Brassica rapa* | 530 | RID70458 |
| *CcMAX1a* | *Cajanus cajanifolius* | 531 | XP_020207800 |
| *CcMAX1b* | *Cajanus cajanifolius* | 534 | XP_020210968 |
| *CsMAX1* | *Cannabis sativa* | 525 | XP_030490461 |
| *CrMAX1* | *Capsella rubella* | 525 | XP_006294008 |
| *CaaMAX1* | *Capsicum annuum* | 532 | XP_016537773 |
| *CaMAX1a* | *Cicer arietinum* | 527 | XP_004498873 |
| *CaMAX1b* | *Cicer arietinum* | 543 | XP_004501462 |
| *CisMAX1* | *Citrus sinensis* | 547 | XP_006466891 |
| *CcMAX1a* | *Citrus clementina* | 538 | XP_006450866 |
| *CcMAX1b* | *Citrus clementina* | 547 | XP_006425560 |
| *CusMAX1* | *Cucumis sativus* | 529 | XP_004141322 |
| *EgMAX1* | *Eucalyptus grandis* | 542 | XP_010047358 |
| *EsMAX1* | *Eutrema salsugineum* | 527 | XP_006408861 |
| *FvMAX1* | *Fragaria vesca* | 531 | XP_004291053 |
| *GrMAX1* | *Gossypium raimondii* | 539 | XP_012442761 |
| *LjMax1* | *Lotus japonicus* | 538 | BBM90835 |
| *NnMAX1* | *Nelumbo nucifera* (*sacred lotus*) | 544 | XP_010262061 |
| *MeMAX1* | *Manihot esculenta* | 527 | XP_021598065 |
| *EgMAX1* | *Erythranthe guttata* | 526 | XP_012857912 |
| *MaMAX1a* | *Musa acuminata* | 529 | XP_009380454 |
| *MaMAX1b* | *Musa acuminata* | 526 | XP_009408870 |
| *ObMAX1* | *Oryza brachyantha* | 543 | XP_006646245 |
| *PdMAX1a* | *Phoenix dactylifera* | 529 | XP_008793212 |
| *PdMAX1b* | *Phoenix dactylifera* | 545 | XP_008782508 |
| *PmMAX1a* | *Prunus mume* | 538 | XP_008220493 |
| *PmMAX1b* | *Prunus mume* | 541 | XP_008220494 |
| *PpMAX1a* | *Prunus persica* | 538 | XP_007222310 |
| *PpMAX1b* | *Prunus persica* | 533 | XP_007224581 |
| *PpMAX1c* | *Prunus persica* | 536 | XP_007225050 |
| *MdMAX1a* | *Malus domestica* | 526 | XP_008357300 |
| *MdMAX1b* | *Malus domestica* | 542 | RXH72971 |
| *MdMAX1c* | *Malus domestica* | 540 | XP_008393629 |
| *MdMAX1d* | *Malus domestica* | 532 | XP_028955031 |
| *VvMAX1* | *Vitis vinifera* | 530 | XP_002279086 |
| *CpMAX1* | *Carica papaya* | 538 | XP_021907713 |
| *RcMAX1* | *Ricinus communis* | 540 | XP_002516084 |
| *SiMAX1a* | *Setaria italica* | 533 | XP_004969659 |
| *SiMAX1b* | *Setaria italica* | 536 | XP_004969660 |
| *SiMAX1c* | *Setaria italica* | 549 | XP_004951458 |
| *GmMAX1a* | *Glycine max* | 551 | AQY54419 |
| *GmMAX1b* | *Glycine max* | 548 | AQY54420 |
| *GmMAX1c* | *Glycine max* | 532 | XP_003549345 |
| *GmMAX1d* | *Glycine max* | 538 | XP_003544542 |
| *PgMAX1* | *Picea glauca* | 544 | AGI65359 |
| *SaMAX1* | *Sinapis alba* | 529 | KAF8102838 |
| *TcMAX1* | *Theobroma cacao* | 539 | XP_007012311 |
| *CmMAX1* | *Cucurbita moschata* | 531 | XP_022936422 |
| *TtMAX1a* | *Triticum turgidum subsp. durum* | 536 | VAH72491 |
| *TtMAX1b* | *Triticum turgidum subsp. durum* | 530 | VAI09923 |
| *TaMAX1a* | *Triticum aestivum* | 518 | KAF7096775 |
| *TaMAX1b* | *Triticum aestivum* | 534 | KAF7025803 |
| *TaMAX1c* | *Triticum aestivum* | 518 | KAF7102706 |
| *TaMAX1d* | *Triticum aestivum* | 536 | KAF6994362 |
| *TaMAX1e* | *Triticum aestivum* | 530 | KAF7045862 |
| *TaMAX1f* | *Triticum aestivum* | 530 | KAF7102157 |
| *TaMAX1g* | *Triticum aestivum* | 516 | KAF7041259 |
| *AetMAX1* | Aegilops tauschii | 529 | XP_020163444 |
| *BdMAX1a* | *Brachypodium distachyon* | 530 | XP_003560652 |
| *BdMAX1b* | *Brachypodium distachyon* | 528 | XP_003575594 |
| *BdMAX1c* | *Brachypodium distachyon* | 531 | XP_003571126 |
| *BdMAX1d* | *Brachypodium distachyon* | 525 | XP_010237353 |
| *BdMAX1e* | *Brachypodium distachyon* | 534 | XP_003562092 |
| *HvMAX1a* | *Hordeum vulgare* | 524 | KAE8788859 |
| *HvMAX1b* | *Hordeum vulgare* | 533 | KAE8810993 |
| *HvMAX1c* | *Hordeum vulgare* | 537 | BAJ97619 |
| *HvMAX1d* | *Hordeum vulgare* | 562 | KAE8781561 |
| *HvMAX1e* | *Hordeum vulgare* | 526 | KAE8781562 |
| *MtMAX1a* | *Medicago truncatula* | 529 | AGI65361 |
| *MtMAX1b* | *Medicago truncatula* | 541 | AGI65360 |
| *SbMAX1a* | *Sorghum bicolor* | 547 | XP_002458367 |
| *SbMAX1b* | *Sorghum bicolor* | 545 | XP_002456213 |
| *SbMAX1c* | *Sorghum bicolor* | 545 | XP_002453551 |
| *SbMAX1d* | *Sorghum bicolor* | 540 | XP_002438586 |
| *MlMAX1a* | *Miscanthus lutarioriparius* | 539 | CAD6237236 |
| *MlMAX1b* | *Miscanthus lutarioriparius* | 521 | CAD6237234 |
| *MlMAX1c* | *Miscanthus lutarioriparius* | 540 | CAD6247547 |
| *MlMAX1d* | *Miscanthus lutarioriparius* | 528 | CAD6336534 |
| *KnMAX1a* | *Klebsormidium nitens* | 537 | GAQ86604 |
| *KnMAX1b* | *Klebsormidium nitens* | 569 | GAQ88536 |
| *KnMAX1c* | *Klebsormidium nitens* | 574 | GAQ88955 |
| *KnMAX1d* | *Klebsormidium nitens* | 550 | GAQ88956 |
| *SfMAX1* | *Sphagnum fallax* | 503 | Sphfalx0047s0130 |

**Supplementary Table 7.** Accession numbers of SOTs used for the phylogenetic analysis in Figure 3. The amino acid sequences are downloadable from NCBI. The functions of some plant SOTs have been identified before [12].

| **Gene** | **Species** | **Size (a.a.)** | **Accession numbers** | **Reference** |
| --- | --- | --- | --- | --- |
| *AtSOT13* | *Arabidopsis thaliana* | 324 | NP_178472 | [13] |
| *AtSOT8* | *Arabidopsis thaliana* | 331 | NP_172799 | [14] |
| *AtSOT14* | *Arabidopsis thaliana* | 347 | NP_196317 | [15] |
| *AtSOT15* | *Arabidopsis thaliana* | 359 | NP_568177 | [15] |
| *AtSOT5* | *Arabidopsis thaliana* | 323 | NP_190093 | [13] |
| *pFST3* | *Flaveria chlorifolia* | 312 | P52836 | [16] |
| *pFST4'* | *Flaveria chlorifolia* | 320 | P52837 | [16] |
| *AtSOT10* | *Arabidopsis thaliana* | 333 | NP_179098 | [17] |
| *AtSOT12* | *Arabidopsis thaliana* | 326 | NP_178471 | [17] |
| *AtSOT16* | *Arabidopsis thaliana* | 338 | NP_177550 | [18] |
| *AtSOT17* | *Arabidopsis thaliana* | 346 | NP_173294 | [18] |
| *AtSOT18* | *Arabidopsis thaliana* | 350 | NP_177549 | [18] |
| *AtTPST* | *Arabidopsis thaliana* | 500 | NP_001320287 | [19] |
| *PtSOT1* | *Populus trichocarpa* | 350 | XP_002318367 | [20] |
| *pBFST3* | *Flaveria bidentis* | 312 | P52835 | [21] |
| *BnST1* | *Brassica napus* | 323 | XP_013723132 | [22] |
| *BnST2* | *Brassica napus* | 324 | AAC63112 | [22] |
| *BnST3* | *Brassica napus* | 325 | XP_013606782 | [22] |
| *BnST4* | *Brassica napus* | 323 | NP_001302500 | [22; 23] |
| *LGS1* | *Sorghum bicolor* | 452 | KAG0530922 | [24] |
| *MlSOT* | *Miscanthus lutarioriparius* | 401 | CAD6255761 |  |
| *TaSOT* | *Triticum aestivum* | 345 | KAF7005357 |  |
| *ZmSOT* | *Zea mays* | 451 | XP_008672387 |  |
| *SxtSULT* | *Microseira wollei* | 302 | ACG63834 | [25] |
| *CyrJ* | *Cylindrospermopsis raciborskii* | 261 | WP_007357796 | [26] |
| *FgSULT1* | *Fusarium graminearum* PH-1 | 307 | XP_011322917 | [2] |
| *FvSULT* | *Fusarium verticillioides* | 309 | RBR08306 | [2] |
| *XlSULT* | *Xylaria longipes* | 553 | RYC60899 | [2] |
| *SULT1A1* | *Homo sapiens* | 295 | NP_001046 | [2] |
| *HsSULT* | *Homo sapiens* | 411 | NP_004798 | [2] |
| *MmSULT* | *Mus musculus* | 411 | NP_056633 | [2] |

**Reference:**

[1] Wakabayashi, T., Hamana, M., Mori, A., Akiyama, R., Ueno, K., Osakabe, K., Osakabe, Y., Suzuki, H., Takikawa, H., Mizutani, M., and Sugimoto, Y. (2019). Direct conversion of carlactonoic acid to orobanchol by cytochrome P450 CYP722C in strigolactone biosynthesis. *Sci. Adv.* 5, eaax9067. doi: 10.1126/sciadv.aax9067

[2] Xie, L., Xiao, D., Wang, X., Wang, C., Bai, J., Yue, Q., Yue, H., Li, Y., Molnár, I., Xu, Y., Zhang, L., and Mitchell Aaron, P. (2020). Combinatorial biosynthesis of sulfated benzenediol lactones with a phenolic sulfotransferase from *Fusarium graminearum* PH-1. *mSphere* 5, e00949-20. doi: 10.1128/mSphere.00949-20

[3] Yoneyama, K., Mori, N., Sato, T., Yoda, A., Xie, X., Okamoto, M., Iwanaga, M., Ohnishi, T., Nishiwaki, H., Asami, T., Yokota, T., Akiyama, K., Yoneyama, K., and Nomura, T. (2018). Conversion of carlactone to carlactonoic acid is a conserved function of MAX1 homologs in strigolactone biosynthesis. *New Phytol.* 218, 1522-1533 .doi: 10.1111/nph.15055

[4] Abe, S., Sado, A., Tanaka, K., Kisugi, T., Asami, K., Ota, S., Kim, H. I., Yoneyama, K., Xie, X., Ohnishi, T., Seto, Y., Yamaguchi, S., Akiyama, K., Yoneyama, K., and Nomura, T. (2014). Carlactone is converted to carlactonoic acid by MAX1 in *Arabidopsis* and its methyl ester can directly interact with AtD14 in vitro. *Proc. Natl. Acad. Sci. U. S. A.* 111, 18084-18089. doi: 10.1073/pnas.1410801111

[5] Wakabayashi, T., Ishiwa, S., Shida, K., Motonami, N., Suzuki, H., Takikawa, H., Mizutani, M., and Sugimoto, Y. (2021). Identification and characterization of sorgomol synthase in sorghum strigolactone biosynthesis. *Plant Physiol.* 185, 902-913.doi: 10.1093/plphys/kiaa113

[6] Yoda, A., Mori, N., Akiyama, K., Kikuchi, M., Xie, X., Miura, K., Yoneyama, K., Sato-Izawa, K., Yamaguchi, S., Yoneyama, K., Nelson, D. C., and Nomura, T. (2021). Strigolactone biosynthesis catalyzed by cytochrome P450 and sulfotransferase in sorghum. *New Phytol.* doi: 10.1111/nph.17737

[7] Cunningham, F. X., Jr., and Gantt, E. (2005). A study in scarlet: enzymes of ketocarotenoid biosynthesis in the flowers of Adonis aestivalis. *Plant J.* 41, 478-492. doi: 10.1111/j.1365-313X.2004.02309.x

[8] Wu, S., Ma, X., Zhou, A., Valenzuela, A., Zhou, K., and Li, Y. (2021). Establishment of strigolactone-producing bacterium-yeast consortium. *Sci. Adv.* 7, eabh4048. doi: 10.1126/sciadv.abh4048

[9] Alberti, S., Gitler, A.D., and Lindquist, S. (2007). A suite of Gateway cloning vectors for high-throughput genetic analysis in *Saccharomyces cerevisiae*. *Yeast* 24, 913-919. doi: 10.1002/yea.1502

[10] Xu, S., Chen, C., and Li, Y. (2020). Engineering of phytosterol-producing yeast platforms for functional reconstitution of downstream biosynthetic pathways. *ACS Synth. Biol.* 9, 3157-3170. doi: 10.1021/acssynbio.0c00417

[11] Entian K.-D., Kötter P. (2007). Twenty five yeast genetic strain and plasmid collections. *Methods Microbiol*. 36, 629–666. doi: https://doi.org/10.1016/S0580-9517(06)36025-4

[12] Hirschmann, F., Krause, F., and Papenbrock, J. (2014). The multi-protein family of sulfotransferases in plants: composition, occurrence, substrate specificity, and functions. *Front. Plant Sci.* 5, 556. doi: 10.3389/fpls.2014.00556

[13] Hashiguchi, T., Sakakibara, Y., Hara, Y., Shimohira, T., Kurogi, K., Akashi, R., Liu, M.-C., and Suiko, M. (2013). Identification and characterization of a novel kaempferol sulfotransferase from *Arabidopsis thaliana*. *Biochem. Biophys. Res. Commun.* 434, 829-835. doi: https://doi.org/10.1016/j.bbrc.2013.04.022

[14] Hashiguchi, T., Sakakibara, Y., Shimohira, T., Kurogi, K., Yamasaki, M., Nishiyama, K., Akashi, R., Liu, M.-C., and Suiko, M. (2013). Identification of a novel flavonoid glycoside sulfotransferase in *Arabidopsis thaliana*. *J. Biochem.* 155, 91-97. doi: 10.1093/jb/mvt102

[15] Gidda, S. K., Miersch, O., Levitin, A., Schmidt, J., Wasternack, C., and Varin, L. (2003). Biochemical and molecular characterization of a hydroxyjasmonate sulfotransferase from *Arabidopsis thaliana*. *J. Biol. Chem.* 278, 17895-17900. doi: 10.1074/jbc.M211943200

[16] Varin, L., DeLuca, V., Ibrahim, R. K., and Brisson, N. (1992). Molecular characterization of two plant flavonol sulfotransferases. *Proc. Natl. Acad. Sci. U. S. A.* 89, 1286-1290. doi: 10.1073/pnas.89.4.1286

[17] Marsolais, F., Boyd, J., Paredes, Y., Schinas, A. M., Garcia, M., Elzein, S., and Varin, L. (2007). Molecular and biochemical characterization of two brassinosteroid sulfotransferases from *Arabidopsis*, AtST4a (At2g14920) and AtST1 (At2g03760). *Planta* 225, 1233-1244. doi: 10.1007/s00425-006-0413-y

[18] Piotrowski, M., Schemenewitz, A., Lopukhina, A., Muller, A., Janowitz, T., Weiler, E. W., and Oecking, C. (2004). Desulfoglucosinolate sulfotransferases from *Arabidopsis thaliana* catalyze the final step in the biosynthesis of the glucosinolate core structure. *J. Biol. Chem.* 279, 50717-50725. doi: 10.1074/jbc.M407681200

[19] Komori, R., Amano, Y., Ogawa-Ohnishi, M., and Matsubayashi, Y. (2009). Identification of tyrosylprotein sulfotransferase in *Arabidopsis*. *Proc. Natl. Acad. Sci. U. S. A.* 106, 15067-15072. doi: 10.1073/pnas.0902801106

[20] Lackus, N. D., Muller, A., Krober, T. D. U., Reichelt, M., Schmidt, A., Nakamura, Y., Paetz, C., Luck, K., Lindroth, R. L., Constabel, C. P., Unsicker, S. B., Gershenzon, J., and Kollner, T. G. (2020). The occurrence of sulfated salicinoids in poplar and their formation by sulfotransferase1. *Plant Physiol.* 183, 137-151. doi: 10.1104/pp.19.01447

[21] Ananvoranich, S., Varin, L., Gulick, P., and Ibrahim, R. (1994). Cloning and regulation of flavonol 3-sulfotransferase in cell-suspension cultures of *Flaveria bidentis*. *Plant Physiol.* 106, 485-491. doi: 10.1104/pp.106.2.485

[22] Rouleau, M., Marsolais, F., Richard, M., Nicolle, L., Voigt, B., Adam, G., and Varin, L. (1999). Inactivation of brassinosteroid biological activity by a salicylate-inducible steroid sulfotransferase from *Brassica napus*. *J. Biol. Chem.* 274, 20925-20930. doi: 10.1074/jbc.274.30.20925

[23] Marsolais, F., Sebastià, C. H., Rousseau, A., and Varin, L. (2004). Molecular and biochemical characterization of BNST4, an ethanol-inducible steroid sulfotransferase from *Brassica napus*, and regulation of *BNST* genes by chemical stress and during development. *Plant Sci.* 166, 1359-1370. doi: https://doi.org/10.1016/j.plantsci.2004.01.019

[24] Gobena, D., Shimels, M., Rich, P. J., Ruyter-Spira, C., Bouwmeester, H., Kanuganti, S., Mengiste, T., and Ejeta, G. (2017). Mutation in sorghum *LOW GERMINATION STIMULANT 1* alters strigolactones and causes *Striga* resistance. *Proc. Natl. Acad. Sci. U. S. A.* 114, 4471-4476. doi: 10.1073/pnas.1618965114

[25] Lukowski, A. L., Denomme, N., Hinze, M. E., Hall, S., Isom, L. L., and Narayan, A. R. H. (2019). Biocatalytic detoxification of paralytic shellfish toxins. *ACS Chem. Biol.* 14, 941-948. doi: 10.1021/acschembio.9b00123

[26] Mejean, A., and Ploux, O. (2021). Biosynthesis of cylindrospermopsin in cyanobacteria: characterization of CyrJ the sulfotransferase. *J. Nat. Prod.* 84, 408-416.doi: 10.1021/acs.jnatprod.0c01089
